# Integration of autophagy-related genes and immune dysregulation reveals a prognostic landscape in multiple myeloma

**DOI:** 10.3389/fonc.2025.1635596

**Published:** 2025-09-17

**Authors:** Yibo Xia, Dong Zheng, Xinyi Zhang, Shuxia Zhu, Enqing Lan, Hansen Ying, Zixing Chen, Bingxin Zhang, Shujuan Zhou, Yu Zhang, Xuanru Lin, Qiang Zhuang, Honglan Qian, Xudong Hu, Yan Zhuang, Qianying Zhang, Xiangjing Zhou, Zuoting Xie, Songfu Jiang, Yongyong Ma, Zhouxiang Jin, Sisi Zheng

**Affiliations:** ^1^ Department of Medical Laboratory Technology, School of Laboratory Medicine and Life Science, Wenzhou Medical University, Wenzhou, Zhejiang, China; ^2^ Department of Hematology, The First Affiliated Hospital of Wenzhou Medical University, Wenzhou, Zhejiang, China; ^3^ Department of Hepatobiliary Surgery, The Second Affiliated Hospital and Yuying Children’s Hospital of Wenzhou Medical University, Wenzhou, Zhejiang, China; ^4^ Department of Blood Transfusion, The First Hospital, Wenzhou Medical College, Wenzhou, Zhejiang, China

**Keywords:** multiple myeloma, autophagy, prognostic landscape, tumor microenvironment, immune dysregulation, drug response

## Abstract

**Background:**

Autophagy is a self-renewal mechanism in which cells degrade damaged organelles or abnormal proteins through lysosomes. This process eliminates harmful components within the cell and maintains energy homeostasis. Multiple myeloma (MM) is a hematological malignancy characterized by uncontrolled plasma cell proliferation. Autophagy plays a dual role in tumorigenesis, yet its prognostic implications in MM remain underexplored.

**Methods:**

Transcriptomic and clinical data from 1,386 MM patients (training cohort: GSE136337, n = 415; validation cohorts: GSE24080, n = 558; GSE4581, n = 413) were analyzed. A seven-gene signature (ATIC, CDKN1A, DNAJB9, EDEM1, GABARAPL1, RAB1A, VAMP7) was identified using LASSO-Cox regression. Predictive performance of the autophagy-related model was assessed via Kaplan-Meier analysis, ROC curves, and nomograms. Immune infiltration, drug sensitivity, and functional pathways of the autophagy-related model were evaluated using CIBERSORT, ESTIMATE, and GSEA. The gene expression in the autophagy prognostic model was verified by qRT-PCR in the U266 and RPMI8226 cell lines and blood samples of multiple myeloma patients from the First Affiliated Hospital of Wenzhou Medical University.

**Results:**

The autophagy-related risk score stratified patients into high-risk and low-risk groups with distinct survival outcomes (high-risk HR = 0.391, 95%CI:0.284-0.540, p < 0.001). The model demonstrated robust predictive accuracy (5-year AUC = 0.729) and was independently validated. High-risk patients exhibited elevated immune checkpoint expression (CD48, CD70, BTLA), stromal infiltration, and drug resistance. Functional enrichment linked high-risk profiles to MYC activation and oxidative phosphorylation. Through qRT-PCR, the accuracy of the autophagy-related model has been verified in the U266 and RPMI8226 cell lines, as well as in the blood samples of multiple myeloma patients from the First Affiliated Hospital of Wenzhou Medical University.

**Conclusion:**

This autophagy-related gene signature provides a reliable prognostic tool for MM, highlighting immune dysregulation and therapeutic resistance mechanisms. Its integration with clinical parameters enhances risk stratification and treatment planning.

## 1 Introduction

Multiple myeloma is the second most common hematological malignancy globally, accounting for approximately 10% of all hematological malignancies. (1) Myeloma cells are plasma cells that secrete immunoglobulins, typically IgG or IgA. As plasma cell tumors proliferate, a condition known as monoclonal gammopathy of undetermined significance (MGUS), a pre-cancerous lesion, may develop (2). This can progress to asymptomatic smoldering multiple myeloma (SMM). Within the first five years after diagnosis, SMM progresses to MM at a rate of approximately 10% per year. In the subsequent five years, the progression rate decreases to 3% per year, and thereafter to 1.5% per year, influenced by underlying cytogenetic factors. (3) The hallmark of MM is the abnormal proliferation of malignant plasma cells in the bone marrow, leading to excessive production of monoclonal immunoglobulin or light chains. This results in bone destruction, hypercalcemia, anemia, and renal insufficiency. (4) The interaction between tumor cells and the immune microenvironment plays a crucial role in the disease’s development and progression. (5) Previous research has demonstrated that the development of multiple myeloma (MM) is closely associated with impaired immune surveillance. Key features include compromised antibody production, imbalances in T-cell and natural killer cell populations, disrupted antigen presentation mechanisms, upregulated inhibitory surface ligands, and recruitment of immunosuppressive cells. (6) Immunotherapies for MM encompass autologous stem cell transplantation (ASCT), immunomodulatory drugs such as thalidomide and lenalidomide, monoclonal antibodies like daratumumab and elotuzumab, chimeric antigen receptor T cells, bispecific antibodies, antibody-drug conjugates, and checkpoint inhibitors. (7) Despite advances in immunomodulatory therapies, relapse remains inevitable, underscoring the need for novel prognostic biomarkers.

Autophagy is an evolutionarily conserved cellular self-degradation mechanism that selectively degrades abnormal proteins, damaged organelles and pathogens through the lysosomal pathway to maintain intracellular homeostasis. According to the different substrate transport modes, autophagy is classified into giant autophagy (forming double-membrane autophagosomes to enclose substrates), microautophagy (direct entrapment of lysosomal membranes for phagocytosis), and chaperone-mediated autophagy (CMA, relying on HSC70 to recognize specific proteins). Under physiological conditions, autophagy is involved in embryonic development, metabolic adaptation and aging regulation. Under pathological conditions, its dysfunction is closely related to neurodegenerative diseases (such as Alzheimer’s disease), tumorigenesis and immune abnormalities. However, its role in cancer remains controversial; it can either inhibit tumor growth by promoting cell cycle arrest, genomic integrity, and reducing necrosis and inflammation (8) or support tumor progression by enhancing cell survival, metastasis, and adaptation to nutritional stress, potentially contributing to metastatic recurrence. In the context of adaptive immune responses, autophagy plays a pivotal role in maintaining the homeostasis, function, and differentiation of T lymphocytes, as well as the survival and development of B lymphocytes and plasma cells. (9) Specifically, autophagy facilitates the transition from B lymphocytes to plasma cells by sustaining cellular energy and viability. (10, 11) It also ensures the balance between endoplasmic reticulum stress, differentiation, and antibody production, thereby determining the stability of the immune microenvironment. However, its role in MM progression is context-dependent, with evidence linking autophagy to both tumor suppression and therapy resistance. Current research suggests that mutations in autophagy-related genes can influence cancer development through alterations in energy metabolism and immune regulation, with effects varying based on the type of cancer cells and their developmental stage. In multiple myeloma (MM), autophagy is essential for maintaining the antibody response and supporting long-lived plasma cells, highlighting its immunological significance in MM. (11, 12) Studies have shown that autophagy markers correlate with MM prognosis. (13) Furthermore, inhibiting TGFβ signaling in bone marrow fibroblasts can disrupt the autophagy pathway that facilitates tumor cell survival, thus overcoming bortezomib resistance in MM patients. (14) A study mentioned that autophagy inhibitor Chloroquine has been found to enhance carfilzomib-induced apoptosis in myeloma cells both *in vitro* and *in vivo*. (15, 16).

Recent studies highlight autophagy-related genes (ARGs) as potential prognostic markers in solid tumors (6), yet their utility in MM remains unclear. This study aims to: (1) identify ARGs associated with MM survival, (2) construct a prognostic model validated across independent cohorts, and (3) elucidate interactions between autophagy, immune infiltration, and drug response.

## 2 Materials and methods

### 2.1 Data collection and processing

Three transcriptomic profiles of multiple myeloma (MM) cohorts—GSE136337, GSE24080, and GSE4581—were downloaded from the Gene Expression Omnibus (GEO, https://www.ncbi.nlm.nih.gov/geo/) along with corresponding detailed clinicopathological information. For downstream analyses, GSE136337 was used as the training cohort, while GSE24080 and GSE4581 served as independent validation cohorts. The clinical characteristics of all study cohorts are summarized in **Table 1**.

**Table 1:** Clinical covariates of the training and validation cohorts.

Characteristics	Training cohort	Validation cohort	Validation cohort
	GSE136337	GSE24080	GSE4581
	(n=415)	(n=558)	(n=413)
Sex			
Female	158 (38%)	222 (40%)	–
Male	257 (62%)	336 (60%)	–
Age			
≤65 years	297 (72%)	422 (76%)	–
>65 years	118 (28%)	136 (24%)	–
Alb			
≥3.5 g/dL	331 (80%)	482 (86%)	–
<3.5 g/dL	84 (20%)	76 (14%)	–
β2M			
<3.5 mg/L	187 (45%)	319 (57%)	–
3.5–5.5 mg/L	109 (26%)	120 (22%)	–
≥5.5 mg/L	119 (29%)	119 (21%)	–
LDH			
≤250 U/L	392 (94%)	509 (91%)	–
>250 U/L	23 (6%)	49 (9%)	–
Del (17p)			
False	411 (99%)	–	–
True	4 (1%)	–	–
t (4,14)			
False	401 (97%)	–	–
True	14 (3%)	–	–
t (14,16)			
False	414 (100%)	–	–
True	1 (0%)	–	–
ISS			
I	163 (39%)	288 (52%)	–
II	133 (39%)	156 (28%)	–
III	119 (29%)	114 (20%)	–
R-ISS			
I	83 (20%)	–	–
II	267 (64%)	–	–
III	65 (16%)	–	–
Risk score			
High	207 (50%)	279 (50%)	206 (50%)
Low	208 (50%)	279 (50%)	207 (50%)
Survival			
Alive	239 (58%)	387 (69%)	334 (81%)

Alb albumin, β2M β2-microglobulin, LDH lactate dehydrogenase.

All three datasets were generated using the Affymetrix Human Genome U133 Plus 2.0 microarray platform, ensuring consistency in gene annotation and probe mapping. To minimize potential technical biases arising from differences in sample processing and expression platforms, we first harmonized gene annotations across the datasets. Each dataset was normalized using the Microarray Suite 5.0 (MAS5.0) algorithm and log2-transformed to ensure data comparability. Principal component analysis (PCA) was performed on the combined expression matrix to assess batch effects, which revealed dataset-specific clustering indicative of significant batch-associated variance. To correct for batch effects, the ComBat function from the “sva” R package was applied, using dataset ID as the batch variable and empirical Bayes adjustment. A subsequent PCA demonstrated a substantial reduction in batch-related clustering, indicating successful batch effect correction while preserving underlying biological variability. (**Supplementary Figure S1**).

### 2.2 Collection of autophagy-related genes

A curated list of 232 autophagy-related genes (ARGs) was retrieved from the Human Autophagy Database (HADb; https://www.autophagy.lu/), a literature-based repository cataloging genes and proteins implicated in autophagy-related biological pathways. To ensure cross-cohort and cross-platform compatibility, we retained only the ARGs that were detectable across all three microarray datasets (GSE136337, GSE24080, and GSE4581), all of which were generated using the Affymetrix Human Genome U133 Plus 2.0 platform. Genes with missing expression values of samples were excluded. After this preprocessing, a total of 209 autophagy-related genes were retained for subsequent analysis.

### 2.3 Screening of autophagy-related genes associated with overall survival in multiple myeloma

To evaluate the prognostic relevance of autophagy-related genes (ARGs) in multiple myeloma (MM), the GSE136337 cohort (n = 415) served as the training cohort. ARGs linked to overall survival (OS) were screened via univariate Cox regression analysis, with statistical significance defined as P < 0.001.

### 2.4 Construction and validation of ARG-related prognostic signature for MM patients

To prevent overfitting of the prognostic risk model, we employed LASSO-penalized Cox regression on the training dataset to identify the most significant autophagy-related genes (ARGs) associated with overall survival (OS). (17) These candidate genes were subjected to multivariate Cox proportional hazards regression analysis, with variables selected stepwise based on the Akaike Information Criterion (AIC). (18) Finally, the risk score calculation for optimizing the prognostic features was as follows:


Risk score=∑ni Coefi * Ai

To evaluate the prognostic relevance of autophagy-related genes (ARGs) in multiple myeloma (MM), the GSE136337 cohort (n = 415) served as the training cohort. ARGs linked to overall survival (OS) were screened via univariate Cox regression analysis, with statistical significance defined as P < 0.001.In this study, the regression coefficient (Coef) corresponds to each autophagy-related gene (ARG) feature (i), with A representing the relative expression of individual ARGs in the signature and “n” denoting the total gene count. Patients were categorized into high-risk and low-risk subgroups based on median risk score thresholds. Cross-dataset comparison of ARG expression profiles was visualized through heatmaps generated using the “ggrisk” R package. Survival disparities between subgroups were analyzed via Kaplan-Meier curves with log-rank testing, while the time-dependent ROC curve quantified the signature’s predictive capacity. To further validate accuracy, risk scores were compared between deceased and surviving patients. External validation was performed using independent MM cohorts (GSE24080, n = 558; GSE4581, n = 413).

### 2.5 Gene interaction networks and genetic alteration profiles of ARGs

To characterize genomic alterations in hematological malignancies, mutational profiles were analyzed using cBioPortal (http://www.cbioportal.org/), with mutation frequencies and chromosomal locations derived from the Gene Set Cancer Analysis (GSCA) framework. Protein-protein interaction (PPI) networks involving autophagy-related genes (ARGs) were constructed via the STRING database (v11.5; https://string-db.org/), revealing functional associations and molecular interplay. Pairwise correlations among ARGs were visualized using the “corrplot” R package. Additionally, risk score-associated ARG expression patterns were validated against the Cancer Cell Line Encyclopedia (CCLE) (https://portals.broadinstitute.org/ccle) to ensure consistency across MM models.

### 2.6 Comparative analysis of clinical characteristics and drug sensitivity among different subgroups

To identify independent prognostic factors influencing overall survival (OS), univariate and multivariate Cox regression analyses were performed across both training and validation cohorts. Subgroup heterogeneity was further examined through comparative analyses of clinical features and therapeutic response variations among molecular subtypes. High-risk cytogenetic abnormalities (HRCAs), including del(17p), amp(1q), t(4;14), t(14;20), t(14;16), and MYC aberrations, were defined based on prior evidence and training cohort data, with chromosomal alterations confirmed via fluorescent *in situ* hybridization (FISH) or cytogenetic analysis. (19) Patients were stratified into three risk cohorts based on cytogenetic profiles: those harboring ≥1 high-risk cytogenetic abnormality (HRCA) constituted the high-risk group; individuals with specific alterations [del(13q), del(16q), del(1p), del(1q), t(11;14), or t(12;14)] were classified as the low-risk subgroup; and patients lacking these abnormalities formed the non-mutation cohort. Drug susceptibility disparities between cohorts were evaluated using the ONCOPREDICT computational framework, a machine learning-driven platform that integrates drug response data from the Genomics of Drug Sensitivity in Cancer (GDSC) database (www.cancerrxgene.org). This framework systematically generates sensitivity scores to quantify predicted therapeutic efficacy across diverse anticancer agents. Differential drug activity between high-risk and low-risk cohorts was prioritized to identify potential therapeutic vulnerabilities.

### 2.7 Tumor-immune microenvironment landscape and potential implications for immunotherapy defined by the signature

CIBERSORT was utilized to estimate the abundance of 22 immune cell types within the complex gene expression profiles of multiple myeloma (MM) patients, (20) including seven types of T cells, naïve and memory B cells, plasma cells, and NK cells, in both the high- and low-risk groups. Samples with a P-value < 0.05 were selected for further analysis. Additionally, we employed the ESTIMATE algorithm to assess immune infiltration levels across different groups. Based on gene expression data and cytogenetic characteristics, we inferred the levels of infiltrating stromal and immune cells in tumor tissues and evaluated tumor purity among the groups. Furthermore, we examined differences in immune checkpoint reactivity between the high-risk and low-risk groups, enabling us to assess the potential effectiveness of targeted therapies in the context of our autophagy-based prognostic model.

### 2.8 Gene set enrichment analysis

To investigate the biological mechanisms underlying the autophagy-related risk signature, Gene Ontology (GO) and Kyoto Encyclopedia of Genes and Genomes (KEGG) pathway analyses were performed. Patients were dichotomized into high- and low-risk subgroups using the median risk score as the cutoff. Gene Set Enrichment Analysis (GSEA v4.3.2; http://www.broad.mit.edu/gsea/) was implemented to identify significantly enriched pathways, with statistical significance defined as a nominal P-value <0.05 and a false discovery rate (FDR) <0.25. (21) Functional annotation of autophagy-associated prognostic features was further validated through Metascape, an integrative bioinformatics platform for gene enrichment analysis.

### 2.9 Establishing a predictive nomogram

A multivariate prognostic nomogram incorporating age, International Staging System (ISS) stage, and autophagy-related risk score was developed using the “rms” package in R. Internal validation of the nomogram’s predictive accuracy was performed through calibration curve analysis. To comparatively assess prognostic performance, time-resolved ROC analyses (1-, 3-, and 5-year survival) were conducted using the “timeROC” package, evaluating the discrimination capacity of the Revised International Staging System (R-ISS), autophagy-related risk scores, and the composite nomogram. Clinical decision-making utility was quantified via Decision Curve Analysis (DCA) implemented with the “ggDCA” package, which estimates net survival benefit by weighting true-positive predictions against false-positive rates across threshold probabilities.

### 2.10 Cell lines and cell culture

U266 and RPMI8226 cells were commercially sourced from Fenghui Biotechnology Co., Ltd. (Hunan, China). The cells were cultured in RPMI-1640 medium (Gibco, Shanghai, China) supplemented with 10% fetal bovine serum (FBS), 0.1 mg/mL streptomycin, and 100 U/mL penicillin G. Cultures were maintained in a humidified atmosphere at 37°C with 5% CO2.

### 2.11 Patients

The study cohort comprised 25 newly diagnosed multiple myeloma patients recruited from the Department of Clinical Hematology at the First Affiliated Hospital of Wenzhou Medical University, with diagnoses confirmed through 2014 International Myeloma Working Group (IMWG) diagnostic criteria. Bone marrow aspirates from these patients and 11 age-matched healthy donors (serving as controls) underwent molecular characterization through standardized PCR protocols. All biological samples were collected under institutional oversight, with written informed consent obtained in accordance with the Declaration of Helsinki guidelines. This protocol received formal ethical approval from the hospital’s Institutional Review Board, ensuring compliance with international biomedical research standards.

### 2.12 RNA extraction, reverse transcription, and quantitative real-time PCR

Total RNA was extracted from bone marrow samples using the Righton DNA & RNA Blood and Tissue Kit (Righton Bio, Shanghai, China) according to the manufacturer’s instructions. Reverse transcription was performed with the cDNA Synthesis Kit (Vazyme, Nanjing, China) to generate cDNA. Quantitative real-time PCR (qRT-PCR) was conducted to detect the expression levels of ARGs using Taq Pro Universal SYBR qRT-PCR Master Mix (Vazyme, Nanjing, China), with β-ACTIN serving as an internal control. Relative expression levels were calculated using the comparative threshold cycle (Ct) method. A complete list of primers used is provided in **Table 2**. All experimental measurements were performed with triplicate technical replicates to maintain methodological consistency and validate data reproducibility.

**Table 2:** Gene primers.

Gene symbol	Polarity	Sequence 5’-3’
ATIC	forward	TTGGAGACTAGACGCCAGTTA
	reverse	GGCATCTGAGATACGCCTTTG
CDKN1A	forward	TGTCCGTCAGAACCCATGC
	reverse	AAAGTCGAAGTTCCATCGCTC
DNAJB9	forward	TCTTAGGTGTGCCAAAATCGG
	reverse	TGTCAGGGTGGTACTTCATGG
EDEM1	forward	GCTACGACAACTACATGGCTC
	reverse	GACTTGGACGGTGGAATCTTT
GABARAPL1	forward	ATGAAGTTCCAGTACAAGGAGGA
	reverse	GCTTTTGGAGCCTTCTCTACAAT
RAB1A	forward	AGATTAAAAAGCGAATGGGTCCC
	reverse	GCTTGACTGGAGTGCTCTGAAT
VAMP7	forward	GAGGTTCCAGACTACTTACGGT
	reverse	GACACTTGAGAACTCGCTATTCA

### 2.13 Statistical analyses

Statistical analyses were executed according to data distribution characteristics: parametric continuous variables underwent two-tailed Student’s t-test for dual-group comparisons, while non-parametric counterparts were evaluated using Mann-Whitney U tests. Categorical variables were assessed through Chi-squared Test. Multi-group comparisons employed ANOVA with *post-hoc* LSD testing for normally distributed datasets, whereas non-Gaussian distributed variables were analyzed via Kruskal-Wallis H-test followed by Dunn’s multiple comparison correction. Bivariate associations were quantified using Pearson’s linear correlation for parametric variables and Spearman’s rank-order correlation for non-parametric measures. Analytical procedures implemented through SPSS 26.0 (IBM Corp) and GraphPad Prism 9.0.

## 3 Results

### 3.1 Sample selection and clinical pathological characteristics

**Figure 1:**
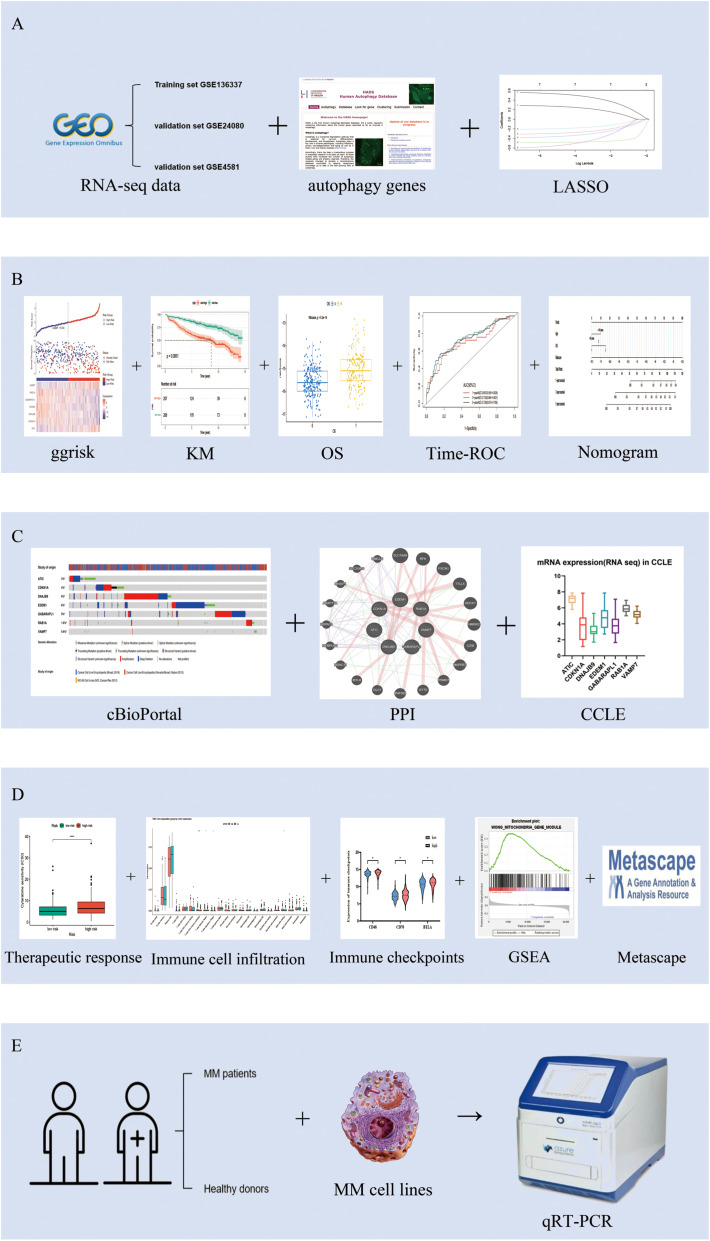
The flowchart of the research design. **(A)** Obtain gene expression data and clinical information of MM patients from the GEO database, and acquire 232 autophagy-related genes from the autophagy website. Combine univariate Cox regression analysis with the LASSO Cox algorithm to develop autophagy-related gene signatures for prognosis. **(B)** Validate the prognostic and predictive capabilities of the model. **(C)** Characterize the gene features of the model. **(D)** Explore the immune and gene enrichment functions of the model. **(E)** Conduct external validation of the model.

**Figure 1** illustrates the study’s schematic workflow. An autophagy-related prognostic model was established through transcriptomic profiling of the GSE136337 cohort, with subsequent validation performed in two independent datasets (GSE24080, n=558; GSE4581, n=413). The combined analytical framework encompassed 1,386 subjects (GSE136337, n=415), whose demographic and clinicopathological parameters (**Table 1**) enabled multivariate Cox regression modeling. This multi-cohort validation strategy ensured rigorous assessment of model generalizability across heterogeneous patient populations.

### 3.2 Construction and verification of autophagy model

**Figure 2:**
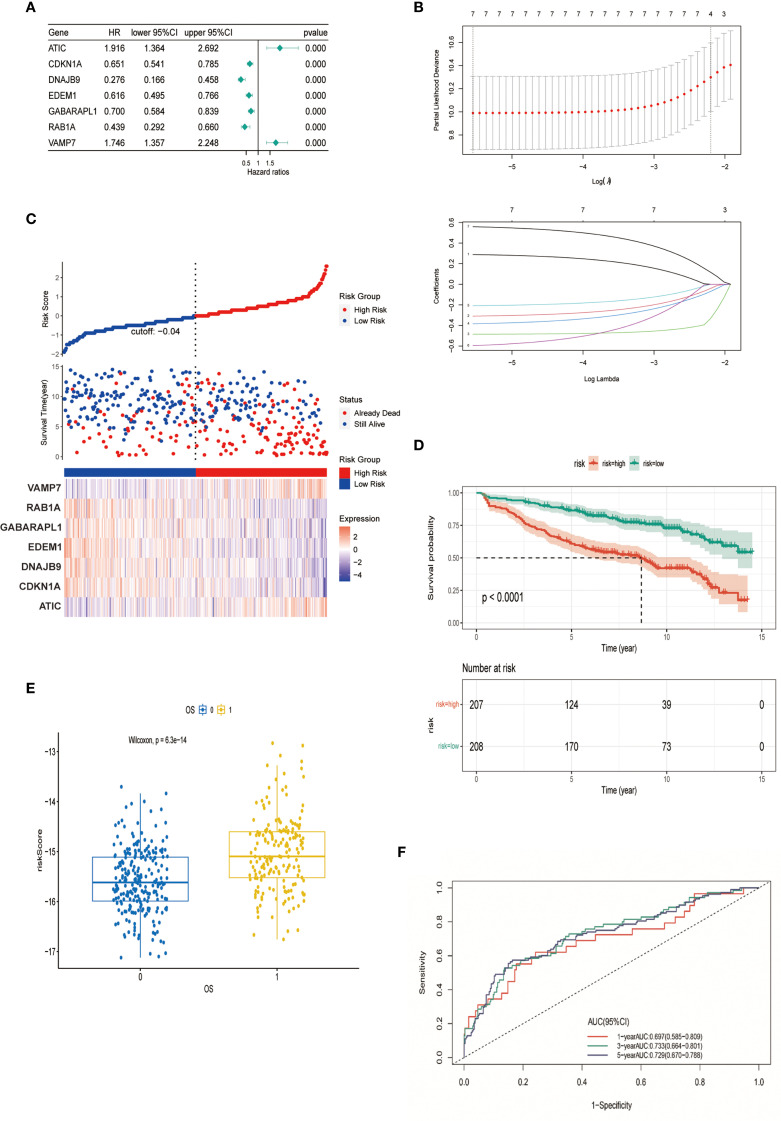
Construction and verification of autophagy model. **(A)** Forest plot representing the prognostic value of autophagy-related genes. **(B)** LASSO Cox regression analysis for variable selection. **(C)** Distribution of survival outcomes and expression of prognostic genes in different cohorts. **(D)** Kaplan-Meier curves for patients in high-risk and low-risk groups (p < 0.0001). **(E)** Scatter plot of the relationship between autophagy risk score and survival outcome. **(F)** AUC of the autophagy model evaluated by time-dependent ROC curve.

Through integrated analysis of the GSE136337 cohort (n=415), seven autophagy-related genes (ARGs) exhibiting significant prognostic associations with overall survival in multiple myeloma patients were rigorously identified via univariate Cox regression followed by LASSO regularization (p<0.001). Based on these findings, an autophagy risk model was constructed (**Figures 2A, B**). The formula for calculating the Autophagy Risk Score is as follows:

Autophagy Risk Score = (0.2889 * expression of ATIC) - (0.3092 * expression of CDKN1A) – (0.4849 * expression of DNAJB9) – (0.3834 * expression of EDEM1) -(0.2079 * expression of GABARAPL1) - (0.5959 * expression of RAB1A) + (0.5588 * expression of VAMP7). Stratification of the cohort into high-risk and low-risk groups was performed using median-based risk score partitioning, revealing a positive correlation between ascending risk scores and cumulative mortality rates. Differential expression profiling of the seven prognostic autophagy-related genes (ARGs) demonstrated distinct risk-group specificity: VAMP7 and ATIC exhibited significant overexpression in high-risk patients, whereas RAB1A, GABARAPL1, EDEM1, DNAJB9, and CDKN1A showed predominant low-risk group enrichment (**Figure 2C**). To evaluate the predictive performance of the model, Kaplan-Meier analysis demonstrated that patients in the high-risk group had significantly shorter overall survival compared to those in the low-risk group (P < 0.001) (**Figure 2D**). The risk score used for constructing the prediction model was significantly correlated with patient OS (p = 6.3e-14) (**Figure 2E**). The model’s discriminative performance was validated through time-dependent ROC analysis, demonstrating robust prognostic capability with area under the curve (AUC) values of 0.697 (1-year OS), 0.733 (3-year OS), and 0.729 (5-year OS) (**Figure 2F**). These metrics confirm the model’s temporal consistency in survival prediction across short-, intermediate-, and long-term clinical endpoints.

### 3.3 The interaction network and genetic variation map of autophagy-related genes

**Figure 3:**
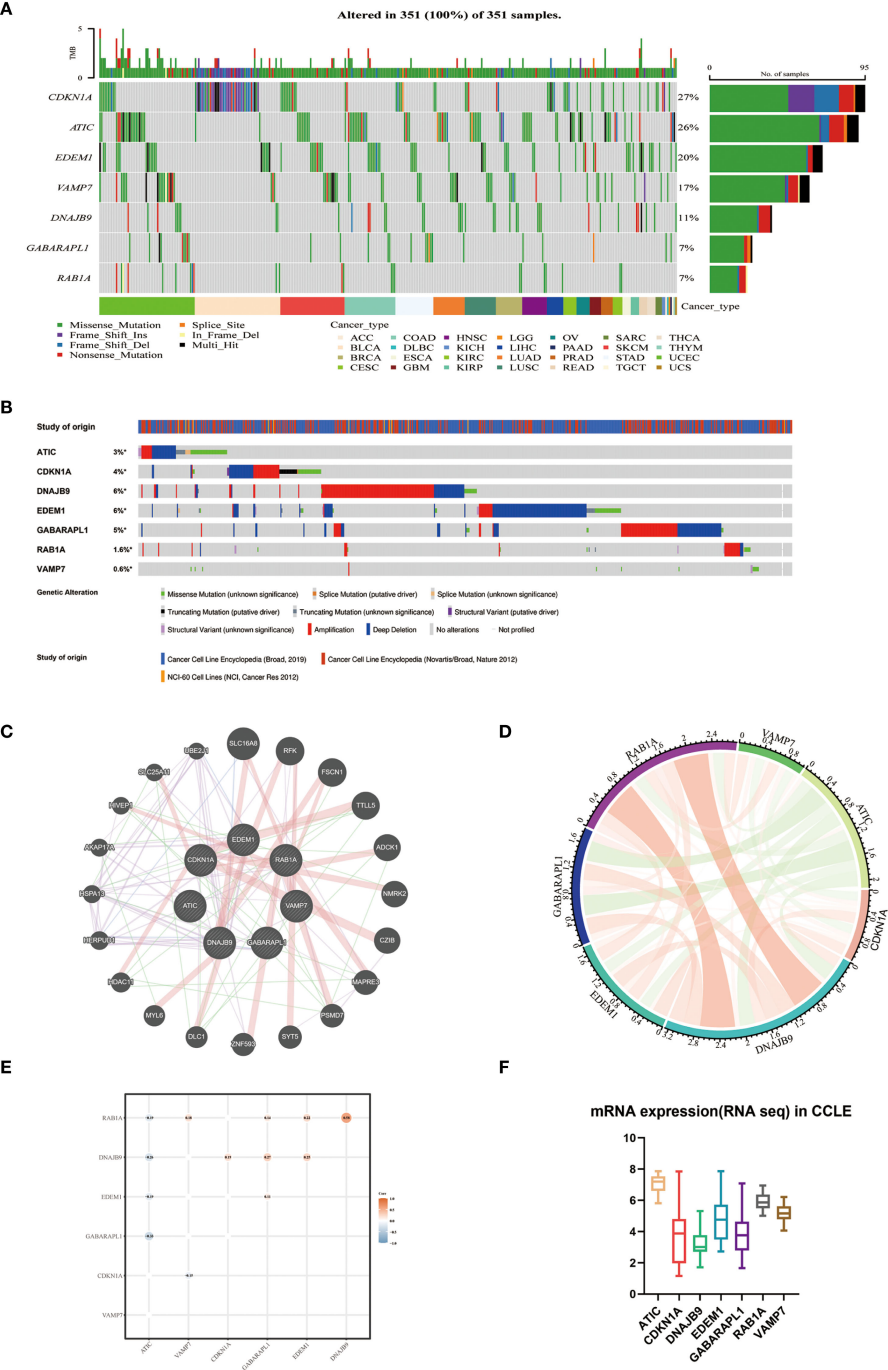
The interaction network and genetic variation map of autophagy-related genes. **(A)** The gene mutation landscape of the autophagy prognostic model derived from TCGA-MM. **(B)** SNV and CNV of 7 genes using cBioPortal for Cancer Genomics. **(C)** Protein-protein interaction network of 7 genes and other closely related proteins. **(D)** Correlation of 7 autophagy genes. **(E)** Co-expression analysis of the characteristics of 7 autophagy genes (GSE136337, n = 424). **(F)** External validation of the expression levels of 7 genes using CCLE.

Based on The Cancer Genome Atlas (TCGA) data, we investigated the mutation landscape of seven genes involved in the autophagy-related models. Our analysis revealed missense mutations as the predominant alteration type, with single nucleotide polymorphisms (SNPs) exhibiting higher prevalence than insertions or deletions (**Figure 3A**). Leveraging the cBioPortal platform, we further characterized the genomic landscape of these seven genes by profiling single nucleotide variations (SNVs) and copy number variations (CNVs) (**Figure 3B**). The alteration frequencies in cancer cell lines were as follows: CDKN1A (4%), DNAJB9 (6%), GABARAPL1 (5%), RAB1A (1.6%), and VAMP7 (0.6%), with amplification being the most common type of alteration. Additionally, ATIC and EDEM1 had mutation frequencies of 3% and 6%, respectively, with deletions being the predominant change. These findings were consistent with our previous data. Furthermore, protein-protein interaction (PPI) networks and co-expression matrices revealed significant relationships among the seven genes (**Figures 3C–E**). In the Cancer Cell Line Encyclopedia (CCLE) database, ATIC was found to be overexpressed, while CDKN1A, DNAJB9, EDEM1, and GABARAPL1 exhibited lower expression levels, aligning with the gene expression patterns observed in our model equations (**Figure 3F**).

### 3.4 Analysis of comparative comparison of clinical characteristics and therapeutic responses among subgroups

Univariate Cox regression analysis indicated that the autophagy risk score, age, albumin, β2-microglobulin (β2M), lactate dehydrogenase (LDH), International Staging System (ISS), and Revised International Staging System (R-ISS) were associated with overall survival (OS) in multiple myeloma (MM) patients. Through multivariable Cox regression analysis adjusted for clinicopathological covariates, the autophagy-related risk signature, chronological age, and International Staging System (ISS) classification emerged as independent prognostic determinants of survival outcomes in the cohort (**Table 3**). Specifically, the autophagy risk score was independently associated with survival rate. In the GSE136337 dataset (n = 415), the hazard ratio (HR) for the autophagy risk score was 0.391(95% CI: 0.284-0.540, p < 0.001). In the GSE24080 dataset (n = 558), the HR was 0.708 (95% CI: 0.520-0.964, p = 0.028).

**Table 3:** Univariate analysis and multi-variate regression analysis of overall survival in the training and validation cohorts by Cox regression analysis.

Characteristics	Training cohort GSE136337 (N = 415)				Validation cohort GSE24080 (N = 558)			
	Univariate analysis		Multi-variate regression analysis		Univariate analysis		Multi-variate regression analysis	
	Regression coefficient (SE)	p	Hazard ratio (95% CI)	p	Regression coefficient (SE)	p	Hazard ratio (95% CI)	p
Sex (female *vs*. male)	-0.248(0.154)	0.107			−0.03 (0.155)	0.848		
Age (<65 *vs*. >65 years)	0.579(0.155)	<0.001*	1.657(1.218-2.253)	0.001*	0.198 (0.178)	0.267		
Albumin (≥3.5 *vs*. <3.5g/dL)	0.410(0.177)	0.021*			0.653 (0.190)	0.001*		
β2M (<3.5 *vs*. 3.5-5.5 *vs*.≥5.5 mg/L)	0.469(0.091)	<0.001*			0.544 (0.088)	<0.001*		
LDH (≤250 *vs*. >250 U/L)	0.732(0.270)	0.007*			1.347 (0.195)	<0.001*		
Del (17p) (false *vs*. true)	0.098(0.417)	0.814						
t(4,14) (false *vs*. true	0.035(0.455)	0.939						
t(14,16) (false *vs*. true)	0.719(1.004)	0.474						
ISS (I *vs*. II *vs*. III)	0.503(0.095)	<0.001*	1.655(1.268-2.159)	<0.001*	0.558 (0.091)	<0.001*	1.644 (1.369–1.974)	<0.001*
R-ISS (I *vs*. II *vs*. III)	0.595 (0.133)	<0.001*						
Metabolic risk (low *vs*. high)	-1.027(0.162)	<0.001*	0.391(0.284-0.540)	<0.001*	-0.459(0.156)	0.003*	0.708(0.52-0.964)	0.028*

We did not include albumin, β2M and LDH in the multi-variate analysis, because they have collinearity with R-ISS or ISS.

Alb albumin, β2M β2-microglobulin, LDH lactate dehydrogenase.

*p < 0.05.

**Figure 4:**
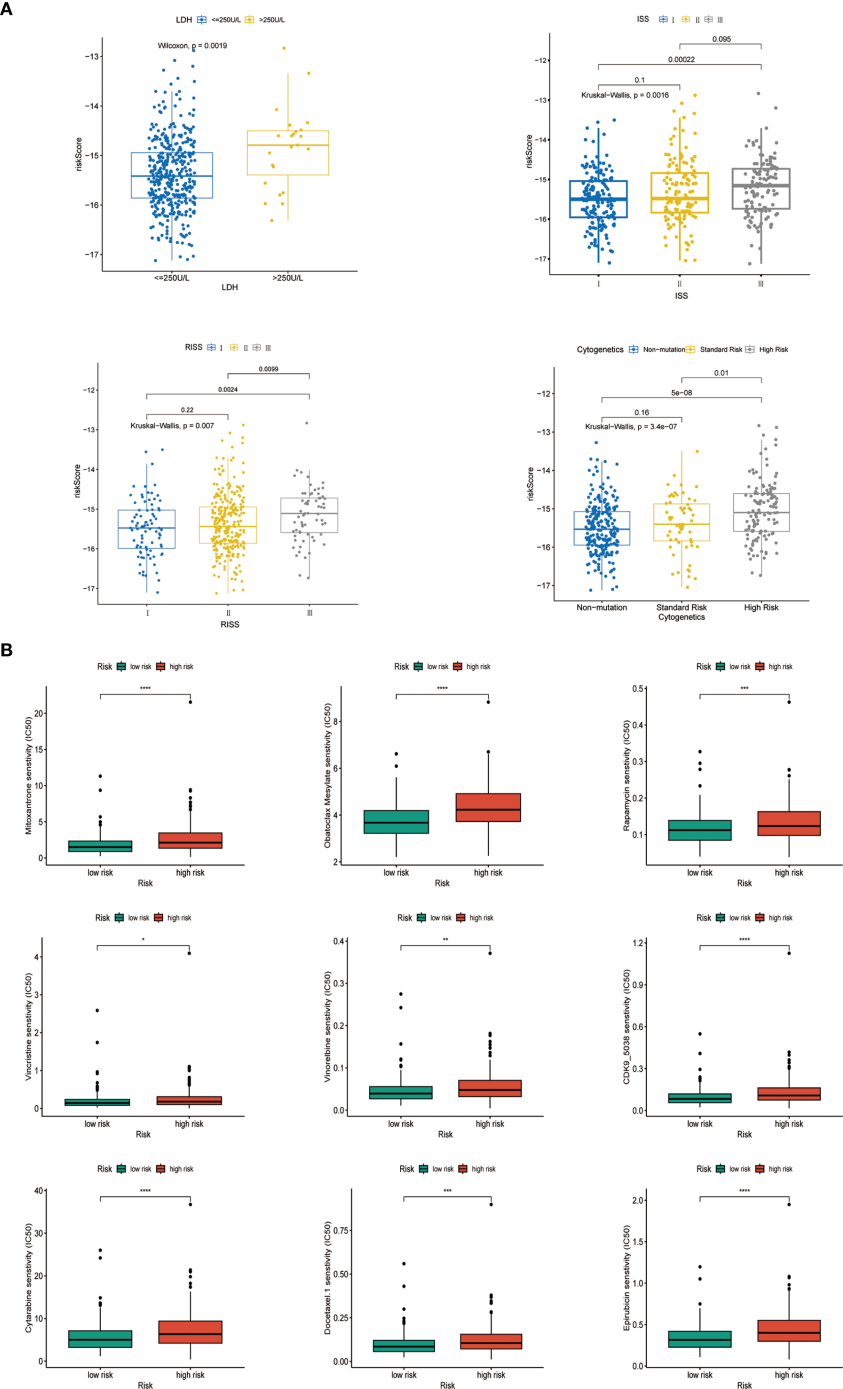
Analysis of comparative comparison of clinical characteristics and therapeutic responses among subgroups. **(A)** The relationship between risk scores and different clinical characteristics (GSE136337, n = 415). **(B)** Evaluation of drug sensitivity among different cohorts (GSE136337, n = 415). ns, insignificant; "*" means "p<=0.05", "**" means "p<=0.01", "***" means "p<=0.001", "****" means "p<=0.0001"

We analyzed the relationships between the characteristics of seven genes in the GSE136337 dataset (n = 415) and various clinicopathological features. The autophagy risk score exhibited a statistically significant elevation in patients with elevated serum lactate dehydrogenase (LDH) levels compared to those with lower LDH concentrations (p<0.001). Furthermore, progressive increments in autophagy risk scores were positively correlated with advancing disease severity as classified by the International Staging System (ISS) and Revised International Staging System (R-ISS), while simultaneously demonstrating a significant association with deteriorating cytogenetic risk profiles. These observations revealed substantial heterogeneity across stratified subgroups, with all comparative analyses reaching statistical significance (**Figure 4A**).

Using the R package ONCOPREDICT, we predicted the IC50 values of 198 drugs for multiple myeloma and compared them between the high- and low-risk groups based on the autophagy risk score. Nine drugs, including CDK9 inhibitors, cytarabine, docetaxel, epirubicin, mitoxantrone, Obatoclax Mesylate, rapamycin, vincristine, and synthetic analogs of vincristine, exhibited lower IC50 values in both groups. These findings highlight the therapeutic potential of the evaluated agents within our autophagy-based prognostic framework. Pharmacogenomic profiling revealed significantly elevated IC50 values in high-risk patients versus low-risk counterparts (p<0.01), with the former demonstrating intrinsic drug resistance and the latter showing enhanced therapeutic sensitivity (**Figure 4B**).

### 3.5 The potential correlation of characteristics in the tumor immune microenvironment

**Figure 5:**
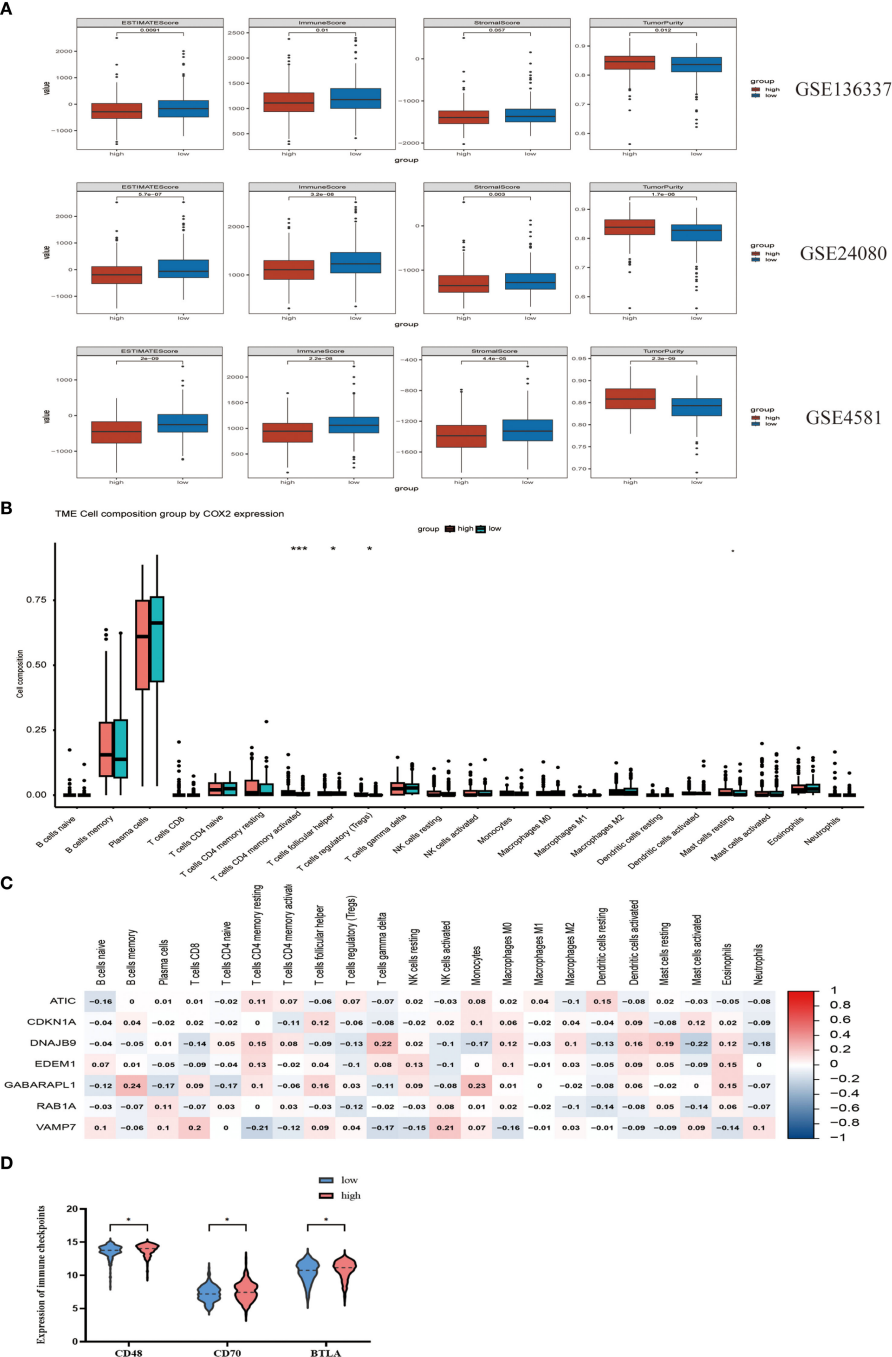
The potential correlation of characteristics in the tumor immune microenvironment. **(A)** Evaluate the differences in immune infiltration between the high-risk and low-risk cohorts in GSE136337, GSE24080 and GSE4581 using the ESTIMATE method. **(B)** Assess the differences in immune infiltration among subgroups based on the CIBERSORT algorithm. **(C)** The relationship between seven autophagy-related prognostic genes and immune cells. **(D)** Comparison of the expression of three immune checkpoints between the high-risk group and the low-risk group. "*" means "p<=0.05", "**" means "p<=0.01", "***" means "p<=0.001", "****" means "p<=0.0001"

To explore the association of autophagy-related risk stratification with tumor microenvironment characteristics in multiple myeloma (MM), we employed the ESTIMATE algorithm to comparatively assess immune score, stromal score, ESTIMATE score, and tumor purity between low- and high-risk cohorts. Our analysis revealed that the immune cell infiltration level was significantly higher in the low-risk cohort (p < 0.01), while the abundance of stromal cells was greater in the high-risk group (p < 0.05). Additionally, tumor purity was higher in the high-risk group. These findings were validated in the GSE24080 and GSE4581 datasets. (**Figure 5A**) Using the CIBERSORT algorithm, we observed that the high-risk cohort exhibited higher levels of immune cell infiltration, specifically in CD4+ T memory activated cells (p < 0.001), T follicular helper cells (p < 0.05), and regulatory T (Tregs) cells (p < 0.05) (**Figure 5B**). The target genes in the prognostic model showed significant correlations with immune cell infiltration in the high-risk group (**Figure 5C**). We also examined the differences in the expression of immune checkpoint molecules between the high-risk and low-risk groups. The results indicated that CD48, CD70, and BTLA were upregulated in the high-risk group (p < 0.05) (**Figure 5D**). These molecules play vital roles in modulating the immune response against tumors and inhibiting immune cell activity. The observed increase in CD48, CD70, and BTLA in the high-risk group suggests potential targets for immunotherapy intervention.

### 3.6 Functional annotation of autophagy-related prognostic signatures in MM

**Figure 6:**
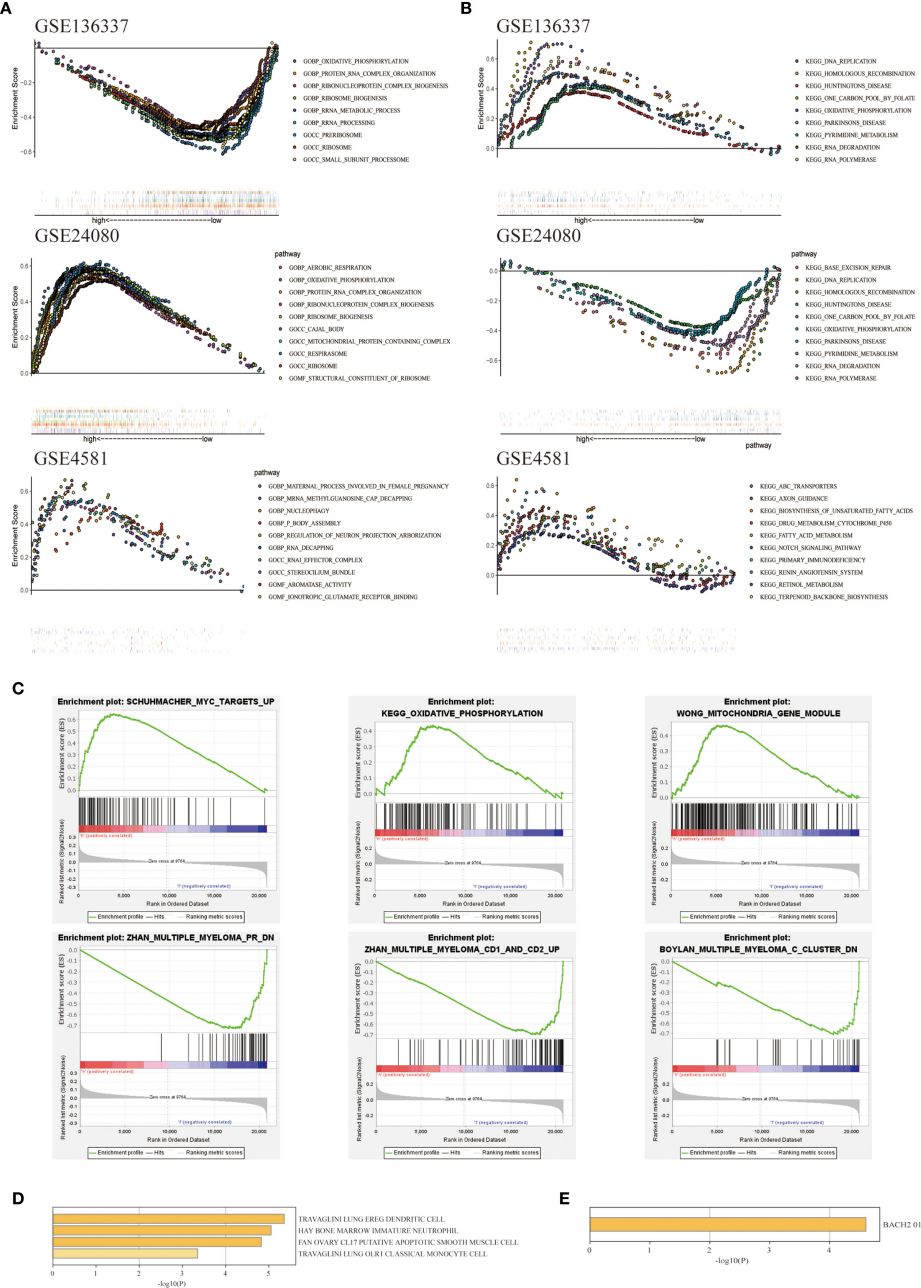
Exploration of biological functions based on prognostic autophagy characteristics. **(A)** GO GSEA enrichment analysis of genes between the two cohorts with NES > 1 in GSE136337, GSE24080 and GSE4581, nominal p-values < 0.05 and FDR < 0.25 are considered statistically significant. **(B)** KEGG GSEA enrichment analysis of genes between the two cohorts with NES > 1 in GSE136337, GSE24080 and GSE4581, nominal p-values < 0.05 and FDR < 0.25 are considered statistically significant. **(C)** Differentially expressed pathways between the two cohorts enriched by GSEA. **(D)** Immune cell types enriched from 7 ARGs obtained from Metascape. **(E)** Pathways enriched in the 7 ARGs obtained from Metascape.

To elucidate the potential mechanisms underlying the autophagy characteristics between the two cohorts, we conducted GO and KEGG on three datasets: GSE136337, GSE24080, and GSE4581. The GSEA analysis was conducted on GSE136337. Gene Ontology (GO) term analysis identified statistically significant enrichment of biological processes associated with autophagic mechanisms and oncogenic pathways (FDR <0.05), including oxidative phosphorylation, nucleophagy, protein-RNA complex assembly, rRNA processing, rRNA metabolic processes, and mitochondrial protein-containing complexes (**Figure 6A**). In the KEGG enrichment analysis, key pathways included oxidative phosphorylation, activation of the notch signaling pathway, base excision repair, RNA degradation, and RNA polymerase (**Figure 6B**). The GSEA analysis indicated that in the high-risk group, pathways were enriched for MYC gene upregulation, oxidative phosphorylation, and altered mitochondrial patterns. In contrast, the low-risk group showed enrichment for downregulated multiple myeloma (MM) pathways, upregulated MM CD1 and CD2 pathways, and downregulated MM C cluster pathways. These pathway changes might contribute to the better prognosis and survival observed in the low-risk cohort. (22) (**Figure 6C**). Using the Metascape database, we conducted enrichment analysis of cell type characteristics, revealing a strong correlation between the features of our predictive model and immature myeloid cells in the bone marrow (**Figure 6D**). Additionally, through the analysis of transcription factor targets, we identified a significant association between BACH2 and the autophagy-related prognostic model features (**Figure 6E**). The transcription factor BACH2 is expressed in various immune cells and plays a crucial role in regulating humoral immune responses and maintaining immune homeostasis. A study showed that Bach2 can regulate the homeostasis of Regulatory T cells and the differentiation of T follicular regulatory cells, which was consistent with the conclusion we reached using the cibersort algorithm (**Figure 5B**), that the infiltration levels of T cells follicular helper (p<0.05) and T cells regulatory (Tregs) (p<0.05) in the high-risk cohort were higher compared to the low-risk cohort, proving the role of autophagy-related prognostic models in immunology.

### 3.7 Construct a predictive nomogram to evaluate clinical applicability

**Figure 7:**
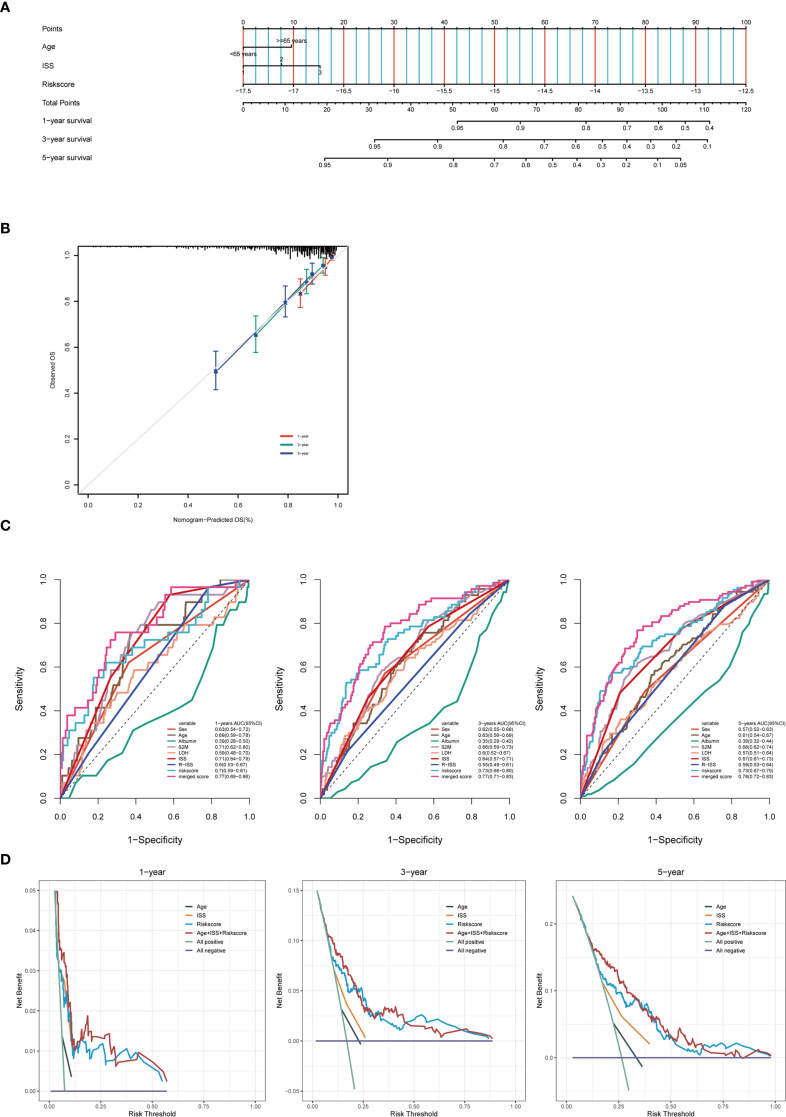
Construct a predictive nomogram to evaluate clinical applicability. **(A)** Training cohort nomogram based on age, ISS stage and autophagy risk score. **(B)** Calibration plots were created to verify the accuracy of 1-year, 3-year and 5-year survival predictions. **(C)** ROC analysis with time-dependent analysis of various clinical covariates for 1, 3 and 5 years. **(D)** DCA was used to determine the net survival benefit and risk score for each clinical feature.

To enhance the efficiency of survival prediction, we developed a combined nomogram model incorporating age, International Staging System (ISS) staging, and autophagy risk score using the GSE136337 dataset (n = 415) (**Figure 7A**). The calibration plot for survival probability demonstrated excellent consistency between predicted and observed values (**Figure 7B**). The area under the curve (AUC) for the merged score at 1-year, 3-year, and 5-year periods were 0.77, 0.77, and 0.78, respectively, which were higher than those of other clinical parameters (**Figure 7C**). This indicates the high predictive accuracy of the autophagy risk score for multiple myeloma patient survival and its significant clinical applicability. When evaluated using decision curve analysis (DCA) (**Figure 7D**), the nomogram’s performance surpassed that of other indicators. Survival analysis demonstrated sustained prognostic accuracy of the autophagy-related risk stratification model across 1-, 3-, and 5-year intervals, confirming its clinical validity as a robust standalone prognostic indicator. These findings position the autophagy risk signature as a biologically informed complement to conventional ISS staging paradigms, providing multidimensional prognostic refinement for personalized risk assessment.

### 3.8 Validation of prognostic features of the external MM cohort

**Figure 8:**
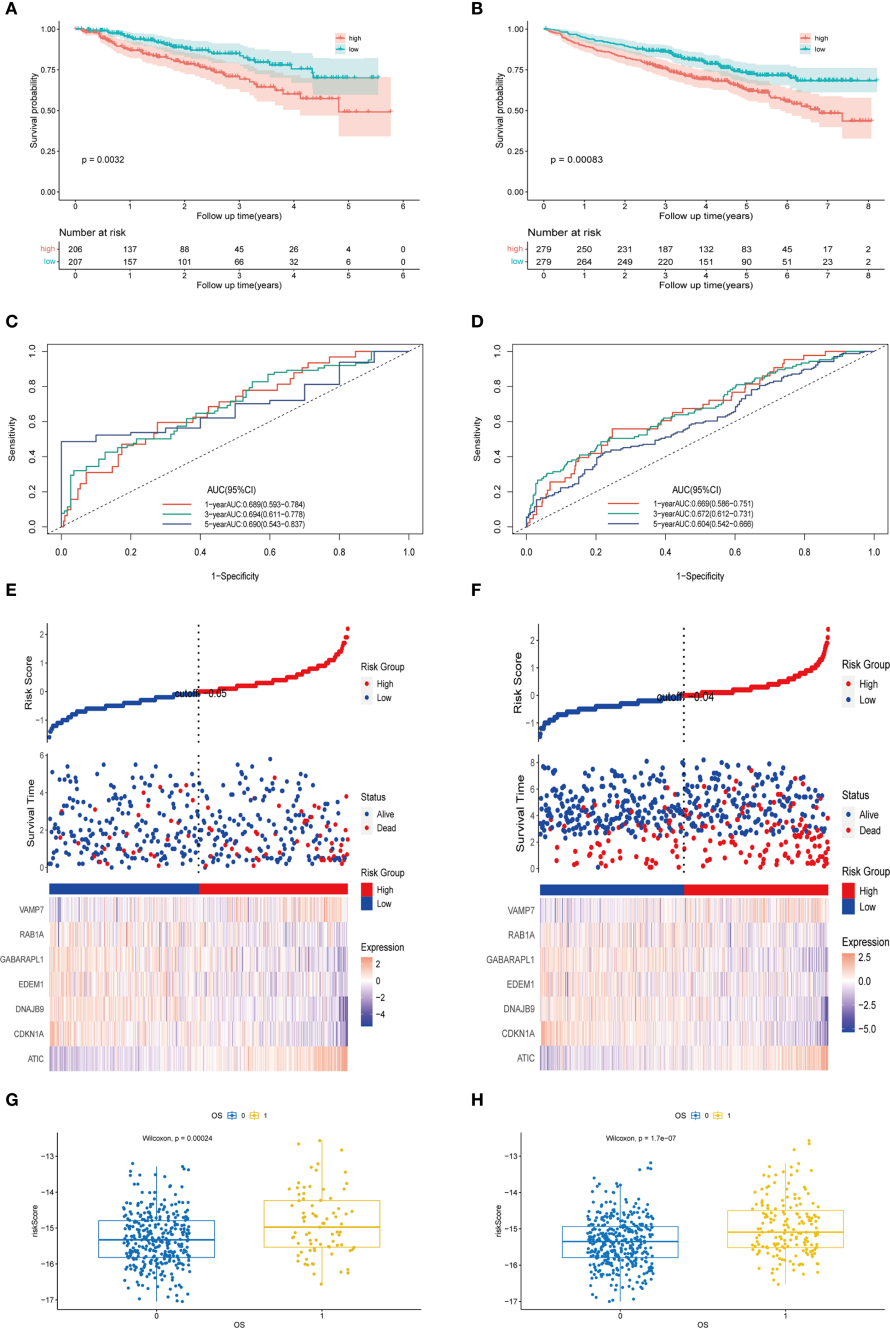
Validation of prognostic features of the external MM cohort. **(A)** The Kaplan-Meier curves of patients in the high-risk and low-risk groups of GSE24080 (p < 0.0001). **(B)** The Kaplan-Meier curves of patients in the high-risk and low-risk groups of GSE4581 (p = 0.0032). **(C)** The AUC of the model evaluated by time-dependent ROC curve in GSE24080. **(D)** The AUC of the model evaluated by time-dependent ROC curve in GSE4581. **(E)** The different expression levels of prognostic genes in the high-risk and low-risk groups and the survival outcomes in GSE24080. **(F)** The different expression levels of prognostic genes in the high-risk and low-risk groups and the survival outcomes in GSE4581. **(G)** The scatter plot of the relationship between autophagy risk score and survival outcome in the high-risk and low-risk groups of GSE24080. **(H)** The scatter plot of the relationship between autophagy risk score and survival outcome in the high-risk and low-risk groups of GSE4581.

To evaluate the predictive efficacy of the autophagy-related prognostic model in external multiple myeloma (MM) cohorts (GSE24080 and GSE4581), we calculated the risk score for each patient using the autophagy feature formula. Patients were stratified into high-risk and low-risk groups based on the median risk score. In the GSE24080 cohort, the Kaplan-Meier (KM) curve demonstrated that patients in the high-risk group had significantly lower survival rates compared to those in the low-risk group (P = 0.0032, **Figure 8A**). The area under the curve (AUC) values for 1-, 3-, and 5-year survival predictions were 0.689, 0.694, and 0.690, respectively (**Figure 8C**). Similarly, in the GSE4581 cohort, patients in the high-risk group exhibited worse prognosis than those in the low-risk group (P = 0.00083, **Figure 8B**). The AUC values for 1-year, 3- year, and 5-year survival predictions were 0.669, 0.672, and 0.604, respectively (**Figure 8D**). The heatmap analysis revealed consistent results with the training cohort: in the validation cohorts, ATIC and VAMP7 expression levels were higher in the high-risk group, while CDKN1A, DNAJB9, EDEM1, GABARAPL1, and RAB1A expression levels were higher in the low-risk group (**Figures 8E, F**). Furthermore, based on the training set GSE136337, the autophagy-related riskscore formula constructed in the validation sets was significantly correlated with both the risk score and overall survival (OS) (**Figures 8G, H**). In words, the findings demonstrated that the autophagy-associated model exhibited independent prognostic capability for predicting overall survival in multiple myeloma patients, showing substantial predictive accuracy.

### 3.9 Carry out external validation using qRT-PCR

**Figure 9:**
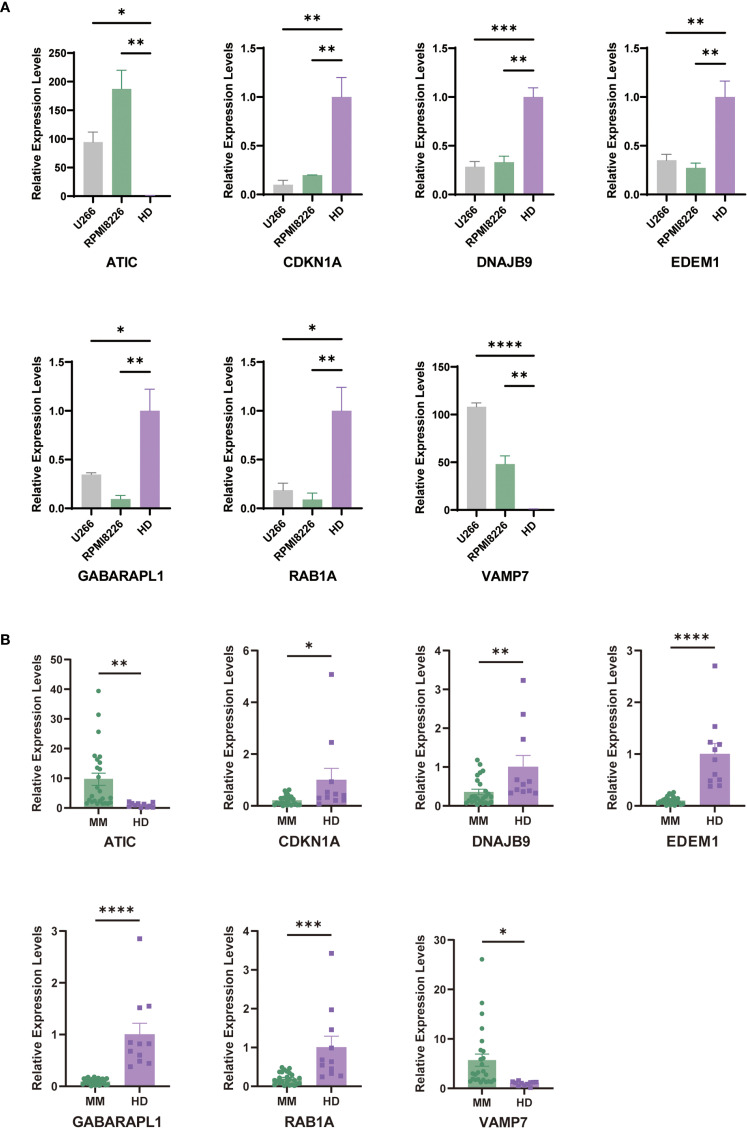
Carry out external validation using qRT-PCR. **(A)** Validation of prognostic ARGs expression in MM cell lines (U266 and RPMI8226) (average ± SEM). **(B)** Comparison of prognostic ARGs expression between MM patients and control samples by qRT-PCR. "*" means "p<=0.05", "**" means "p<=0.01", "***" means "p<=0.001", "****" means "p<=0.0001"

To further validate our predictive model, we conducted PCR assays on multiple myeloma (MM) cell lines and patient samples. The results showed that in MM cell lines (U266 and RPMI8226), the expression levels of ATIC and VAMP7 were significantly higher in the MM group compared to the control group, while the expression levels of CDKN1A, DNAJB9, EDEM1, GABARAPL1, and RAB1A were significantly lower than those in the control group (**Figure 9A**). Consistent with these findings, we also performed PCR assays on human samples. The results indicated that the expression levels of ATIC and VAMP7 were higher in MM patients compared to normal individuals, while the expression levels of CDKN1A, DNAJB9, EDEM1, GABARAPL1, and RAB1A were notably lower in MM patients than in the normal group (**Figure 9B**).

## 4 Discussion

The occurrence and development of tumors necessitate metabolic reprogramming in cancer cells. (23) Oncogenic events driving malignant transformation reprogram metabolic pathways to meet the increased bioenergetic and biosynthetic demands of cancer cells while simultaneously reducing oxidative stress, a process essential for sustaining their proliferation and survival. The tumor microenvironment (TME) can deplete specific nutrients, thereby inducing nutrient clearance mechanisms that sustain cancer cell proliferation, compelling cancer cells to adapt.

Autophagy serves as the primary mechanism responsible for delivering various cellular cargos to lysosomes for degradation and recycling. (24) Autophagy exhibits paradoxical effects on tumor progression; it can inhibit early-stage tumor development yet support the growth and metastasis of established tumors. Multiple preclinical studies have demonstrated that autophagy facilitates the activation of oncogenes and/or the inactivation of tumor suppressors, thereby promoting the growth and metabolism of advanced tumors. (25) For example, selective forms of autophagy enhance cancer cell survival by eliminating damaged mitochondria, thereby reducing the efficacy of chemotherapy-induced acute metabolic stress in eradicating tumor cells. Consequently, targeting autophagy is crucial for addressing tumor progression and treatment resistance. (26) Autophagy also plays a significant role in the tumor microenvironment and associated immune cells. Studies have shown that autophagy in the liver enables regulatory T cells to function, promoting tumor immune tolerance. (27) In a systemic autophagy inhibition model, where a dominant-negative Atg4b mutant was inducibly expressed, acute autophagy blockade in established Kras-driven pancreatic tumors led to substantial tumor regression, suggesting that both host-derived and tumor cell-autonomous autophagy contribute to tumorigenesis. This study underscores the potential of integrating autophagy inhibitors with chemotherapy to mitigate drug resistance during the chemotherapy phase. (28).

A prognostic framework comprising seven autophagy-related genes (ARGs) was constructed via LASSO regression. The model’s reliability was consistently confirmed in two independent validation cohorts, with high-risk patients demonstrating poorer clinical outcomes. Longitudinal ROC curves assessed over time validated the model’s predictive reliability. Additionally, multivariate Cox regression identified autophagy-related signatures as an independent prognostic indicator, outperforming conventional clinical parameters in predictive efficacy.

The high-risk cohort displayed elevated risk parameters, including increased lactate dehydrogenase (LDH) concentrations, advanced International Staging System (ISS) and Revised ISS (RISS) stages, and unfavorable cytogenetic abnormalities. Risk-associated genes in the model were predominantly upregulated in myeloma cases, whereas protective genes exhibited marked overexpression in low-risk individuals. These findings align with previous prognostic studies. Additionally, the high-risk group demonstrated resistance to several therapeutic agents, including CDK9, cytarabine, docetaxel, epirubicin, mitoxantrone, Obatoclax Mesylate, rapamycin, vincristine, and its synthetic analogues, highlighting the potential of the autophagy prognostic model in predicting treatment resistance in multiple myeloma (MM).

GSEA was employed to elucidate the potential mechanisms underlying autophagy-related prognostic biomarkers. Functional enrichment analysis revealed that pathways predominantly enriched in the high-risk group include MYC gene upregulation, oxidative phosphorylation, and aberrant mitochondrial patterns, all of which are closely associated with autophagy and tumor progression. Besides, the low-risk group demonstrated a significant degree of richness in pathways related to MM downregulation, MM CD1 and CD2 upregulation, and MM C cluster downregulation. These observations might be attributed to the relatively favorable prognosis and extended survival period of patients in the low-risk group. These findings clarify the association between poor prognosis in high-risk individuals and autophagy, providing potential molecular insights into how autophagy may influence disease characteristics and progression in multiple myeloma (MM). To further elucidate the connection between autophagy and the specific pathophysiology of multiple myeloma (MM), it is important to emphasize that several hallmark features of MM, including osteolytic bone disease, overproduction of monoclonal protein (M-protein), and the unique bone marrow microenvironment, may intersect with autophagy-related mechanisms. For example, enhanced autophagy may promote osteoclast-mediated bone resorption, thereby contributing to the skeletal complications frequently observed in MM. In addition, autophagy-driven modulation of endoplasmic reticulum stress may facilitate M-protein secretion and support plasma cell survival within the hypoxic bone marrow niche. Although the autophagy-related genes (ARGs) identified in our study did not directly overlap with classical MM driver genes such as MAF translocations, certain metabolic regulators like ATIC may indirectly assist tumor cells in adapting to the bone marrow microenvironment. Further investigations are needed to clarify how specific ARGs influence MM-associated bone destruction, angiogenesis, and the complex crosstalk with immune cells within the marrow ecosystem. Accumulating evidence indicates that the immune microenvironment plays a significant role in the occurrence and development of multiple myeloma. In our study, high-risk individuals exhibited immune dysfunction characterized by lower immune cell counts and higher tumor purity. Specifically, the high-risk cohort demonstrated significantly elevated levels of memory-activated cells (p < 0.001), T follicular helper cells (p < 0.05), and regulatory T cells (Tregs) (p < 0.05). Enrichment analysis using the Metascape database further corroborated the association between our constructed autophagy prognostic model and T follicular helper cells (p < 0.05) as well as Tregs (p < 0.05), thereby validating the role of these features in the immune microenvironment. High-risk individuals exhibited elevated expression levels of immunogenicity and immune checkpoint markers. To ensure the robustness of these findings, we further applied the ESTIMATE algorithm to evaluate tumor purity, stromal content, and immune infiltration. Our results revealed that the high-risk group exhibited significantly higher tumor purity and lower immune infiltration, indicating that the observed immune alterations were not due to non-tumor cell contamination. These findings suggested that the autophagy-related high-risk group may harbor a complex mechanism that contributed to defining the immune microenvironment and predicting the efficacy of immune-targeted therapies. Resistance to proteasome inhibitors and immunomodulatory drugs often involves enhanced autophagy as a protective mechanism. Therefore, the autophagy features identified here may mark cell populations prone to standard therapy escape. Moreover, recent studies highlight the impact of BRAF mutations in multiple myeloma, which activate the MAPK signaling pathway and may induce compensatory autophagy activation. Specifically, MAPK-mediated phosphorylation leads to inactivation of the transcriptional repressor Capicua, which normally suppresses autophagy-dependent survival pathways (29). This suggests that myeloma patients harboring BRAF mutations could exhibit distinct autophagy gene expression profiles and altered responses to therapy. Incorporating mutation status into the autophagy risk model in the future could refine patient stratification and uncover novel therapeutic vulnerabilities related to MAPK-autophagy crosstalk.

To overcome the limitations of single-biomarker approaches in predicting outcomes for multiple myeloma, and in view of its marked biological diversity and heterogeneous clinical trajectories, we established a composite nomogram by systematically integrating training and external validation cohorts. This predictive tool incorporated three critical variables: patient age, autophagy-derived risk stratification, and International Staging System (ISS) classification. Calibration curves revealed enhanced predictive capacity for time-specific survival probabilities compared to isolated parameters. Decision curve analysis (DCA) further substantiated the nomogram’s greater clinical utility relative to conventional prognostic metrics. Furthermore, while we selected the median risk score to define high- and low-risk groups to enhance comparability and avoid overfitting. These findings position the autophagy risk stratification as a complementary prognostic biomarker that effectively augments traditional ISS staging protocols.

In our prognostic model, ATIC and VAMP7 were identified as risk genes, whereas RAB1A, GABARAPL1, EDEM1, DNAJB9, and CDKN1A were classified as protective genes. A large number of studies have shown that these genes are significantly associated with the occurrence and development of cancer. Specifically, the ATIC gene (5-aminomethylimidazole-4-carboxamide ribonucleotide formyltransferase/IMP cyclase), located on chromosome 2q35, encodes a bifunctional enzyme responsible for catalyzing the final two steps of purine biosynthesis, converting imidazole formylamino ribonucleotide to inosine monophosphate. This process is essential for cell proliferation and synthetic metabolism and plays a critical role in metabolic reprogramming for cell survival. ATIC promotes tumor survival, proliferation, and migration by modulating the AMPK-mTOR-S6K1 signaling pathway. (30) High expression levels of ATIC have been linked to poor prognosis in multiple myeloma (MM) and liver cancer. (31, 32) RAB1A, a Rab GTPase, is crucial in the early stages of stress-induced autophagy and participates in autophagosome formation, interacting with both early and late endocytic vesicles. (33, 34) Notably, elevated RAB1A expression has generally been associated with poor tumor prognosis in existing studies. However, in our model, RAB1A functions as a protective gene, as confirmed by PCR validation. This discrepancy may be attributed to the dual nature of autophagy, necessitating further molecular mechanism studies to elucidate its role in different tumor stages. GABARAPL1 participates in various mechanisms such as autophagy, cell death, cell proliferation and tumor progression by mediating the transport of proteins or vesicles. (35) Unlike RAB1A, GABARAPL1 is involved in the later stage of autophagosome formation. (36) A study has shown that GABARAPL1 is regulated by the FoxO transcription factor and can be activated under stress conditions. (37, 38) GABARAPL1 plays a crucial role in selective autophagy. Its interaction with p62 facilitates the autophagic degradation of ubiquitinated protein aggregates, while its association with the mitochondrial-associated protein NIX1 contributes to mitochondrial autophagy. By degrading unnecessary protein aggregates and organelles, GABARAPL1 may play a protective role against tumor progression. (39, 40) Studies have shown that GABARAPL1 expression is downregulated in various cancer cell lines. In breast cancer, neuroblastoma, hepatocellular carcinoma, and lung adenocarcinoma, higher expression levels of GABARAPL1 correlate with reduced risk of tumor metastasis and improved patient survival rates. (19, 41–43) EDEM1 encodes an α-mannosidase-like protein 1, which functions as a putative mannose-binding protein targeting misfolded glycoproteins for ER-associated degradation (ERAD). (44, 45) This pathway bypasses conventional transitional endoplasmic reticulum exit sites and involves budding transport from the rough endoplasmic reticulum via interactions between EDEM1’s transmembrane region and calnexin. (46, 47) Under unstressed conditions, EDEM1 levels within the lumen of the endoplasmic reticulum are minimal. It is sequestered into LC3-I-coated vesicles and rapidly degraded, thereby preventing premature disruption of ongoing protein folding processes and maintaining normal protein synthesis. Proteasome inhibitors can suppress the growth of renal cancer cells by modulating the gene expression of EDEM1, a downstream effector molecule in the unfolded protein response (UPR), through inhibition of GPR78 expression. This suggests that in multiple myeloma (MM), whether proteasome inhibitors such as chloroquine also inhibit tumor progression via this pathway warrants further investigation. (48) Anti-tumor drugs melatonin and valproic acid have been shown to upregulate EDEM1 expression, enhance endoplasmic reticulum stress, and trigger cell death pathways in bladder cancer. (49) In a prognostic model associated with autophagy in female lung adenocarcinoma, EDEM1 exhibits a protective effect. (19) DNAJB9 belongs to the molecular chaperone family of heat shock proteins 40 (Hsp40) and plays a critical role in protein folding, unfolding, translation, degradation, and intracellular signal transduction. Several Hsp40 family members have been confirmed to possess tumor suppressor functions. (50) Historically, research on DNAJB9 has been limited, with most studies focusing on fibrillary glomerulonephritis. (51) It has also been implicated in midbrain longevity in mice. (52)Overexpression of DNAJB9 can inhibit metastasis in triple-negative breast cancer and serves as a protective factor in breast cancer prognosis models. (53, 54) Additionally, it acts as a protective gene in autophagy-related prognosis models for lung adenocarcinoma. (55) CDKN1A/p21 is a cell cycle inhibitor responsible for regulating cell growth and proliferation and is associated with cellular senescence. (56, 57) Numerous studies have demonstrated that CDKN1A functions as a tumor suppressor gene in multiple myeloma (MM). CDKN1A also exhibits tumor suppressor activity in other hematological malignancies. In acute myeloid leukemia (AML), the expression of CDKN1A/p21 is significantly reduced in refractory patients, indicating a poor prognosis. (58) In chronic myeloid leukemia (CML), CDKN1A expression inhibits the invasiveness of CML cells. (59) VAMP7 is a SNARE protein that mediates specific membrane fusion during intracellular transport and maintains mitochondrial homeostasis by regulating autophagy. (60) In an autophagy-related prognostic model for lung adenocarcinoma, VAMP7 has been identified as a risk gene. (19) Previous studies have shown that in AML, knockdown of VAMP7 can inhibit cell growth and increase apoptosis, with lower VAMP7 levels significantly associated with longer overall survival (OS) in AML patients. (61).

However, our study has several limitations that warrant acknowledgment. First, the construction and validation of our prognostic model relied on retrospective data from public databases and clinical samples, necessitating validation in large-scale prospective cohorts to confirm the clinical utility of the identified autophagy-related gene signatures. Secondly, while our analyses suggest mechanistic connections between autophagy regulation and MYC pathway activation, potentially through alterations in autophagic flux that impact mitochondrial quality control and metabolic reprogramming, these relationships require experimental validation. Future studies employing genetic manipulation of key autophagy-related genes in primary MM cells, coupled with assessment of MYC activity and downstream metabolic pathways, will be essential to establish functional evidence for this regulatory axis. Thirdly, although our computational analyses revealed that the high-risk group exhibited significantly higher tumor purity and lower immune infiltration—indicating that observed immune alterations were not artifacts of non-tumor cell contamination—these findings require confirmation through direct experimental approaches such as immunohistochemistry or multiparameter flow cytometry. Finally, the drug sensitivity predictions generated through ONCOPREDICT are based on established cell line profiles and may not fully capture the complexity of the tumor microenvironment or clonal heterogeneity in patient samples. These results should therefore be considered hypothesis-generating and require validation in patient-derived models or clinical cohorts before translation to clinical practice.

## 5 Conclusion

Our findings provide new mechanistic understanding of autophagy’s involvement in multiple myeloma (MM) progression. We observed that distinct autophagy-related molecular patterns correlate with clinical heterogeneity and differential therapeutic responses to both conventional chemotherapy and emerging immunotherapies. Our prognostic model demonstrated robust associations with critical oncogenic processes, particularly immune microenvironment alterations and tumor progression pathways, while maintaining significant prognostic relevance for patient survival outcomes. Through systematic validation, we established the framework’s potential dual utility for prognostic stratification and therapeutic strategy optimization in MM clinical practice.

## Data Availability

The datasets presented in this study can be found in online repositories. The names of the repository/repositories and accession number(s) can be found in the article/**supplementary material**.

## Supplementary material

Supplementary Figure 1 Principal Component Analysis (PCA) before and after batch effect correction using ComBat. **(A)** PCA plot of the merged datasets (GSE136337, GSE24080, GSE4581) before batch effect correction. **(B)** PCA plot after applying ComBat adjustment.

## References

[1] Rajkumar SV. Multiple myeloma: 2020 update on diagnosis, risk-stratification and management. Am J Hematol. (2020) 95:548–67. doi: 10.1002/ajh.25791, PMID: 32212178

[2] Landgren O Kyle RA Pfeiffer RM Katzmann JA Caporaso NE Hayes RB. Monoclonal gammopathy of undetermined significance (MGUS) consistently precedes multiple myeloma: a prospective study. Blood. (2009) 113:5412–7. doi: 10.1182/blood-2008-12-194241, PMID: 19179464 PMC2689042

[3] Rajkumar SV Gupta V Fonseca R Dispenzieri A Gonsalves WI Larson D. Impact of primary molecular cytogenetic abnormalities and risk of progression in smoldering multiple myeloma. Leukemia. (2013) 27:1738–44. doi: 10.1038/leu.2013.86, PMID: 23515097 PMC3773463

[4] Rajkumar SV. Multiple myeloma: 2016 update on diagnosis, risk-stratification, and management. Am J Hematol. (2016) 91:719–34. doi: 10.1002/ajh.24402, PMID: 27291302 PMC5291298

[5] Sperling AS Anderson KC. Facts and hopes in multiple myeloma immunotherapy. Clin Cancer Res. (2021) 27:4468–77. doi: 10.1158/1078-0432.CCR-20-3600, PMID: 33771856 PMC8364865

[6] Rodriguez-Otero P Paiva B Engelhardt M Prosper F San MJ. Is immunotherapy here to stay in multiple myeloma? Haematologica. (2017) 102:423–32. doi: 10.3324/haematol.2016.152504, PMID: 28082344 PMC5394971

[7] Shah UA Mailankody S. Emerging immunotherapies in multiple myeloma. BMJ. (2020) 370:m3176. doi: 10.1136/bmj.m3176, PMID: 32958461

[8] Degenhardt K Mathew R Beaudoin B Bray K Anderson D Chen G. Autophagy promotes tumor cell survival and restricts necrosis, inflammation, and tumorigenesis. Cancer Cell. (2006) 10:51–64. doi: 10.1016/j.ccr.2006.06.001, PMID: 16843265 PMC2857533

[9] Cui B Lin H Yu J Yu J Hu Z. Autophagy and the immune response. Adv Exp Med Biol. (2019) 1206:595–634. doi: 10.1007/978-981-15-0602-4_27, PMID: 31777004 PMC7120363

[10] Conway KL Kuballa P Khor B Zhang M Shi HN Virgin HW. ATG5 regulates plasma cell differentiation. Autophagy. (2013) 9:528–37. doi: 10.4161/auto.23484, PMID: 23327930 PMC3627668

[11] Pengo N Scolari M Oliva L Milan E Mainoldi F Raimondi A. Plasma cells require autophagy for sustainable immunoglobulin production. Nat Immunol. (2013) 14:298–305. doi: 10.1038/ni.2524, PMID: 23354484

[12] Oliva L Cenci S. Autophagy in plasma cell pathophysiology. Front Immunol. (2014) 5:103. doi: 10.3389/fimmu.2014.00103, PMID: 24659989 PMC3950468

[13] Jung G Roh J Lee H Gil M Yoon DH Suh C. Autophagic markers BECLIN 1 and LC3 are associated with prognosis of multiple myeloma. Acta Haematol. (2015) 134:17–24. doi: 10.1159/000368848, PMID: 25871810

[14] Frassanito MA De Veirman K Desantis V Di Marzo L Vergara D Ruggieri S. Halting pro-survival autophagy by TGFbeta inhibition in bone marrow fibroblasts overcomes bortezomib resistance in multiple myeloma patients. Leukemia. (2016) 30:640–8. doi: 10.1038/leu.2015.289, PMID: 26487273

[15] Jarauta V Jaime P Gonzalo O de Miguel D Ramirez-Labrada A Martinez-Lostao L. Inhibition of autophagy with chloroquine potentiates carfilzomib-induced apoptosis in myeloma cells *in vitro* and in *vivo* . Cancer Lett. (2016) 382:1–10. doi: 10.1016/j.canlet.2016.08.019, PMID: 27565383

[16] Amaravadi RK Yu D Lum JJ Bui T Christophorou MA Evan GI. Autophagy inhibition enhances therapy-induced apoptosis in a Myc-induced model of lymphoma. J Clin Invest. (2007) 117:326–36. doi: 10.1172/JCI28833, PMID: 17235397 PMC1765515

[17] Fontanarosa JB Dai Y. Using LASSO regression to detect predictive aggregate effects in genetic studies. BMC Proc. (2011) 5 Suppl 9:S69. doi: 10.1186/1753-6561-5-S9-S69, PMID: 22373537 PMC3287908

[18] Vrieze SI. Model selection and psychological theory: a discussion of the differences between the Akaike information criterion (AIC) and the Bayesian information criterion (BIC). Psychol Methods. (2012) 17:228–43. doi: 10.1037/a0027127, PMID: 22309957 PMC3366160

[19] Liu Z Zhang K Zhao Z Qin Z Tang H. Prognosis-related autophagy genes in female lung adenocarcinoma. Med (Baltimore). (2022) 101:e28500. doi: 10.1097/MD.0000000000028500, PMID: 35029906 PMC8735786

[20] Newman AM Liu CL Green MR Gentles AJ Feng W Xu Y. Robust enumeration of cell subsets from tissue expression profiles. Nat Methods. (2015) 12:453–7. doi: 10.1038/nmeth.3337, PMID: 25822800 PMC4739640

[21] Subramanian A Tamayo P Mootha VK Mukherjee S Ebert BL Gillette MA. Gene set enrichment analysis: a knowledge-based approach for interpreting genome-wide expression profiles. Proc Natl Acad Sci U S A. (2005) 102:15545–50. doi: 10.1073/pnas.0506580102, PMID: 16199517 PMC1239896

[22] Zhan F Huang Y Colla S Stewart JP Hanamura I Gupta S. The molecular classification of multiple myeloma. Blood. (2006) 108:2020–8. doi: 10.1182/blood-2005-11-013458, PMID: 16728703 PMC1895543

[23] Martinez-Reyes I Chandel NS. Cancer metabolism: looking forward. Nat Rev Cancer. (2021) 21:669–80. doi: 10.1038/s41568-021-00378-6, PMID: 34272515

[24] Debnath J Gammoh N Ryan KM. Autophagy and autophagy-related pathways in cancer. Nat Rev Mol Cell Biol. (2023) 24:560–75. doi: 10.1038/s41580-023-00585-z, PMID: 36864290 PMC9980873

[25] Fujii S Mitsunaga S Yamazaki M Hasebe T Ishii G Kojima M. Autophagy is activated in pancreatic cancer cells and correlates with poor patient outcome. Cancer Sci. (2008) 99:1813–9. doi: 10.1111/j.1349-7006.2008.00893.x, PMID: 18616529 PMC11159933

[26] Mathew R White E. Autophagy, stress, and cancer metabolism: what doesn't kill you makes you stronger. Cold Spring Harb Symp Quant Biol. (2011) 76:389–96. doi: 10.1101/sqb.2012.76.011015, PMID: 22442109

[27] Poillet-Perez L Sharp DW Yang Y Laddha SV Ibrahim M Bommareddy PK. Autophagy promotes growth of tumors with high mutational burden by inhibiting a T-cell immune response. Nat Cancer. (2020) 1:923–34. doi: 10.1038/s43018-020-00110-7, PMID: 34476408 PMC8409526

[28] Yang A Herter-Sprie G Zhang H Lin EY Biancur D Wang X. Autophagy sustains pancreatic cancer growth through both cell-autonomous and nonautonomous mechanisms. Cancer Discov. (2018) 8:276–87. doi: 10.1158/2159-8290.CD-17-0952, PMID: 29317452 PMC5835190

[29] Da VM Solimando AG Garitano-Trojaola A Barrio S Munawar U Strifler S. CIC mutation as a molecular mechanism of acquired resistance to combined BRAF-MEK inhibition in extramedullary multiple myeloma with central nervous system involvement. Oncologist. (2020) 25:112–8. doi: 10.1634/theoncologist.2019-0356, PMID: 32043788 PMC7011664

[30] Li M Jin C Xu M Zhou L Li D Yin Y. Bifunctional enzyme ATIC promotes propagation of hepatocellular carcinoma by regulating AMPK-mTOR-S6 K1 signaling. Cell Commun Signal. (2017) 15:52. doi: 10.1186/s12964-017-0208-8, PMID: 29246230 PMC5732395

[31] Zhang H Xia P Liu J Chen Z Ma W Yuan Y. ATIC inhibits autophagy in hepatocellular cancer through the AKT/FOXO3 pathway and serves as a prognostic signature for modeling patient survival. Int J Biol Sci. (2021) 17:4442–58. doi: 10.7150/ijbs.65669, PMID: 34803509 PMC8579461

[32] Li R Chen G Dang Y He R Liu A Ma J. Upregulation of ATIC in multiple myeloma tissues based on tissue microarray and gene microarrays. Int J Lab Hematol. (2021) 43:409–17. doi: 10.1111/ijlh.13397, PMID: 33226193

[33] Gyurkovska V Murtazina R Zhao SF Shikano S Okamoto Y Segev N. Dual function of Rab1A in secretion and autophagy: hypervariable domain dependence. Life Sci Alliance. (2023) 6. doi: 10.26508/lsa.202201810, PMID: 36781179 PMC9939007

[34] Mukhopadhyay A Nieves E Che FY Wang J Jin L Murray JW. Proteomic analysis of endocytic vesicles: Rab1a regulates motility of early endocytic vesicles. J Cell Sci. (2011) 124:765–75. doi: 10.1242/jcs.079020, PMID: 21303926 PMC3039020

[35] Chakrama FZ Seguin-Py S Le Grand JN Fraichard A Delage-Mourroux R Despouy G. GABARAPL1 (GEC1) associates with autophagic vesicles. Autophagy. (2010) 6:495–505. doi: 10.4161/auto.6.4.11819, PMID: 20404487

[36] Weidberg H Shvets E Shpilka T Shimron F Shinder V Elazar Z. LC3 and GATE-16/GABARAP subfamilies are both essential yet act differently in autophagosome biogenesis. EMBO J. (2010) 29:1792–802. doi: 10.1038/emboj.2010.74, PMID: 20418806 PMC2885923

[37] Sengupta A Molkentin JD Paik JH Depinho RA Yutzey KE. FoxO transcription factors promote cardiomyocyte survival upon induction of oxidative stress. J Biol Chem. (2011) 286:7468–78. doi: 10.1074/jbc.M110.179242, PMID: 21159781 PMC3045002

[38] Sengupta A Molkentin JD Yutzey KE. FoxO transcription factors promote autophagy in cardiomyocytes. J Biol Chem. (2009) 284:28319–31. doi: 10.1074/jbc.M109.024406, PMID: 19696026 PMC2788882

[39] Pankiv S Clausen TH Lamark T Brech A Bruun JA Outzen H. p62/SQSTM1 binds directly to Atg8/LC3 to facilitate degradation of ubiquitinated protein aggregates by autophagy. J Biol Chem. (2007) 282:24131–45. doi: 10.1074/jbc.M702824200, PMID: 17580304

[40] Novak I Kirkin V Mcewan DG Zhang J Wild P Rozenknop A. Nix is a selective autophagy receptor for mitochondrial clearance. EMBO Rep. (2010) 11:45–51. doi: 10.1038/embor.2009.256, PMID: 20010802 PMC2816619

[41] Berthier A Seguin S Sasco AJ Bobin JY De Laroche G Datchary J. High expression of gabarapl1 is associated with a better outcome for patients with lymph node-positive breast cancer. Br J Cancer. (2010) 102:1024–31. doi: 10.1038/sj.bjc.6605568, PMID: 20197771 PMC2844027

[42] Roberts SS Mori M Pattee P Lapidus J Mathews R O'Malley JP. GABAergic system gene expression predicts clinical outcome in patients with neuroblastoma. J Clin Oncol. (2004) 22:4127–34. doi: 10.1200/JCO.2004.02.032, PMID: 15483022

[43] Du X Qi Z Xu J Guo M Zhang X Yu Z. Loss of GABARAPL1 confers ferroptosis resistance to cancer stem-like cells in hepatocellular carcinoma. Mol Oncol. (2022) 16:3703–19. doi: 10.1002/1878-0261.13305, PMID: 36062307 PMC9580891

[44] Zuber C Cormier JH Guhl B Santimaria R Hebert DN Roth J. EDEM1 reveals a quality control vesicular transport pathway out of the endoplasmic reticulum not involving the COPII exit sites. Proc Natl Acad Sci U S A. (2007) 104:4407–12. doi: 10.1073/pnas.0700154104, PMID: 17360537 PMC1810509

[45] Oda Y Hosokawa N Wada I Nagata K. EDEM as an acceptor of terminally misfolded glycoproteins released from calnexin. Science. (2003) 299:1394–7. doi: 10.1126/science.1079181, PMID: 12610305

[46] Cali T Galli C Olivari S Molinari M. Segregation and rapid turnover of EDEM1 by an autophagy-like mechanism modulates standard ERAD and folding activities. Biochem Biophys Res Commun. (2008) 371:405–10. doi: 10.1016/j.bbrc.2008.04.098, PMID: 18452703

[47] de Haan CA Molinari M Reggiori F. Autophagy-independent LC3 function in vesicular traffic. Autophagy. (2010) 6:994–6. doi: 10.4161/auto.6.7.13309, PMID: 20814233

[48] Matsumura K Sakai C Kawakami S Yamashita F Hashida M. Inhibition of cancer cell growth by GRP78 siRNA lipoplex via activation of unfolded protein response. Biol Pharm Bull. (2014) 37:648–53. doi: 10.1248/bpb.b13-00930, PMID: 24694610

[49] Liu S Liang B Jia H Jiao Y Pang Z Huang Y. Evaluation of cell death pathways initiated by antitumor drugs melatonin and valproic acid in bladder cancer cells. FEBS Open Bio. (2017) 7:798–810. doi: 10.1002/2211-5463.12223, PMID: 28593135 PMC5458469

[50] Saibil H. Chaperone machines for protein folding, unfolding and disaggregation. Nat Rev Mol Cell Biol. (2013) 14:630–42. doi: 10.1038/nrm3658, PMID: 24026055 PMC4340576

[51] Nasr SH Dasari S Lieske JC Benson LM Vanderboom PM Holtz-Heppelmann CJ. Serum levels of DNAJB9 are elevated in fibrillary glomerulonephritis patients. Kidney Int. (2019) 95:1269–72. doi: 10.1016/j.kint.2019.01.024, PMID: 31010480

[52] Kim H Huh YJ Kim JH Jo M Shin JH Park SC. Identification and evaluation of midbrain specific longevity-related genes in exceptionally long-lived but healthy mice. Front Aging Neurosci. (2022) 14:1030807. doi: 10.3389/fnagi.2022.1030807, PMID: 36711211 PMC9874112

[53] Kim HY Kim YM Hong S. DNAJB9 suppresses the metastasis of triple-negative breast cancer by promoting FBXO45-mediated degradation of ZEB1. Cell Death Dis. (2021) 12:461. doi: 10.1038/s41419-021-03757-x, PMID: 33966034 PMC8106677

[54] Ou YT Cheng WY Zheng T Maurer MA Anastassiou D. Breast cancer prognostic biomarker using attractor metagenes and the FGD3-SUSD3 metagene. Cancer Epidemiol Biomarkers Prev. (2014) 23:2850–6. doi: 10.1158/1055-9965.EPI-14-0399, PMID: 25249324

[55] Zhang F Xie S Zhang Z Zhao H Zhao Z Sun H. A novel risk model based on autophagy pathway related genes for survival prediction in lung adenocarcinoma. Med Sci Monit. (2020) 26:e924710. doi: 10.12659/MSM.924710, PMID: 32873769 PMC7486793

[56] Abbas T Dutta A. p21 in cancer: intricate networks and multiple activities. Nat Rev Cancer. (2009) 9:400–14. doi: 10.1038/nrc2657, PMID: 19440234 PMC2722839

[57] El-Deiry WS. p21(WAF1) mediates cell-cycle inhibition, relevant to cancer suppression and therapy. Cancer Res. (2016) 76:5189–91. doi: 10.1158/0008-5472.CAN-16-2055, PMID: 27635040 PMC5028108

[58] Vitkeviciene A Skliute G Zucenka A Borutinskaite V Navakauskiene R. Potential prognostic markers for relapsed/refractory vs. responsive acute myeloid leukemia. Cancers (Basel). (2022) 14. doi: 10.3390/cancers14112752, PMID: 35681732 PMC9179343

[59] Yao FY Zhao C Zhong FM Qin TY Wen F Li MY. m(6)A Modification of lncRNA NEAT1 Regulates Chronic Myelocytic Leukemia Progression via miR-766-5p/CDKN1A Axis. Front Oncol. (2021) 11:679634. doi: 10.3389/fonc.2021.679634, PMID: 34354942 PMC8329653

[60] Aoyagi K Ohara-Imaizumi M Itakura M Torii S Akimoto Y Nishiwaki C. VAMP7 regulates autophagy to maintain mitochondrial homeostasis and to control insulin secretion in pancreatic beta-cells. Diabetes. (2016) 65:1648–59. doi: 10.2337/db15-1207, PMID: 26953164

[61] Kamachi K Ureshino H Watanabe T Yoshida-Sakai N Fukuda-Kurahashi Y Kawasoe K. Combination of a new oral demethylating agent, OR2100, and venetoclax for treatment of acute myeloid leukemia. Cancer Res Commun. (2023) 3:297–308. doi: 10.1158/2767-9764.CRC-22-0259, PMID: 36860654 PMC9973401

