# Supplementary figures and images for: Integration of autophagy-related genes and immune dysregulation reveals a prognostic landscape in multiple myeloma
Source: Front Oncol. 2025 Sep 17;15:1635596. doi: 10.3389/fonc.2025.1635596 (PMC12483933; doi:10.3389/fonc.2025.1635596)

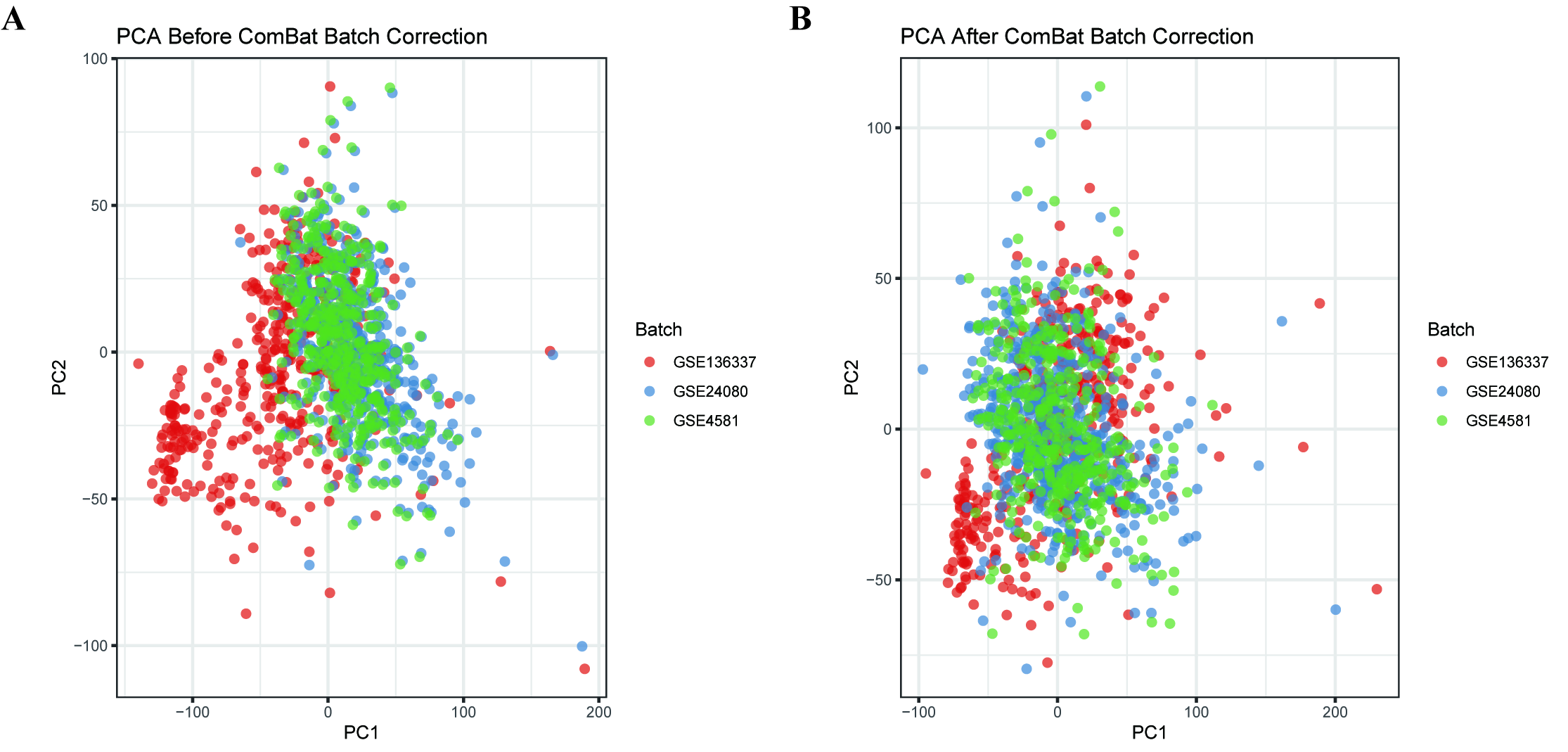

Supplement: Supplementary Figure 1 — Principal Component Analysis (PCA) before and after batch effect correction using ComBat. (A) PCA plot of the merged datasets (GSE136337, GSE24080, GSE4581) before batch effect correction. (B) PCA plot after applying ComBat adjustment. [file Image1.tif]
